# EETANet: A Human-Centered Intelligent Framework for Exercise Posture Quality Assessment

**DOI:** 10.3390/bioengineering13101212

**Published:** 2026-10-17

**Authors:** Esraa Hassan, Amira Samy Talaat, Aliya Aleryani, Rana Albelaihi, M. A. Elsabagh

**Affiliations:** 1 Department of Machine Learning and Information Retrieval, Faculty of Artificial Intelligence, Kafrelsheikh University, Kafrelsheikh 33516, Egypt; 2 Computers and Systems Department, Electronics Research Institute, Cairo 12622, Egypt; 3 Department of Computer Science, College of Computer Science, King Khalid University, Abha 61421, Saudi Arabia; 4 Department of Computer Science, College of Engineering and Information Technology, Onaizah Colleges, Qassim 56447, Saudi Arabia

**Keywords:** human-centered AI, posture analysis, biomechanical signal processing, machine learning, exercise quality, Savitzky–Golay filter, multi-domain feature extraction, ambient intelligence, rehabilitation technology

## Abstract

As physical rehabilitation and fitness monitoring increasingly shift toward autonomous and home-based healthcare, accurate and intelligent posture assessment systems have become essential for supporting personalized rehabilitation and clinical decision making. This study proposes the Edge-Enhanced Temporal Attention Network (EETANet), a human-centered ambient intelligence framework for automated exercise posture assessment and quality evaluation. The proposed framework is designed as an end-to-end computational pipeline that transforms raw multivariate motion signals into posture quality predictions using a compact set of core biomechanical posture features. The pipeline integrates data preprocessing, signal enhancement, multi-domain feature representation, and deep neural classification to capture the relevant spatial and temporal characteristics of human movement. Signal preprocessing incorporates data cleaning, z-score normalization, and Savitzky–Golay smoothing to improve signal quality while preserving temporal dynamics. Subsequently, informative posture representations are extracted through complementary temporal, spectral, and morphological descriptors, enabling the model to characterize motion variations associated with posture quality. These representations are learned using a Multilayer Perceptron (MLP) classifier within the proposed EETANet framework to model nonlinear relationships between posture features and quality levels. Experimental evaluation demonstrates that the compact five-feature configuration achieves an accuracy of 90.83%, precision of 90.99%, recall of 90.83%, and F1-score of 90.84%. Notably, this compact configuration outperforms the corresponding 24-feature MLP configuration, which achieves 88.83% accuracy, indicating that a reduced set of core posture features can provide effective discriminative information while avoiding unnecessary feature redundancy. These findings demonstrate the potential of EETANet as an efficient framework for automated posture quality assessment in rehabilitation, fitness monitoring, and ambient healthcare applications.

## 1. Introduction

Exercise is one of the key pillars of global health and rehabilitation, and its effectiveness is highly reliant on how well the exercise is performed. The biomechanical advantage of exercise is lost, and the risk of musculoskeletal injuries is greatly elevated, due to improper posture during exercise. Physical therapy and fitness training are becoming more prevalent in the home-and-away environment, and so there is an increasing demand for “Ambient Intelligent” systems to provide automated, high-fidelity feedback on the quality of the exercise [1]. Until now, laboratory-type optical motion capture systems have been used to obtain high-accuracy posture analysis.

However, recent developments in human-centered design and wearable sensor technology have made it possible to move towards portable monitoring frameworks. Ensemble deep models can achieve high accuracy in the recognition of posture, even in home settings, which is applicable and achievable in previously non-controlled environments. While these developments are promising, existing systems still must address the complexities of bringing AI and healthcare IoT together. According to to effectively manage complex data streams produced during health monitoring, wearable-based systems need to rely on AI-assisted approaches [2].

One of the solutions being sought to overcome these challenges is to focus on the “Human-centered” development to ensure technology meets user needs. As users increasingly drive the adoption of digital health tools, their success can increasingly rely on designing around the practical challenges of emerging trends [3]. Moreover, the development of multimodal deep learning has enabled the in situ assessment of biomechanical models, which is crucial for sports training and performance analysis and requires low-latency feedback [4].

In this study, we propose a human-centered intelligent posture analysis framework to assess the quality of exercise with a multi-stage pipeline. Our system uses raw multivariate motion signals to make extensive predictions of posture quality ranging from Very Poor to Excellent. Architectures are systematically combined that include high-fidelity preprocessing with Savitzky–Golay filters and multi-domain feature extraction. As shown by [5], a complex signal processing method, integrated with machine learning models like Random Forest and MLP, is key to the correct classification of human activity and proper scientific rigor and usability of postural evaluation. This research makes the following main contributions:

- An integrated EETANet framework for automated exercise posture quality assessment and rehabilitation monitoring.
- Multi-stage signal intelligence pipeline combining temporal features, FFT-based frequency-domain information, and peak detection descriptors.
- Temporal attention mechanisms are designed to capture motion continuity and subtle posture deviations.
- Compact biomechanically meaningful representations, reducing the dependence on many input features.

The novelty of EETANet does not arise from the individual use of established signal processing or machine learning algorithms. Instead, the proposed contribution lies in their structured integration into a human-centered posture quality assessment pipeline and in the formulation of an edge-enhanced temporal representation that complements spectral and morphological descriptors. This design provides a unified mechanism for transforming multivariate motion signals into compact, biomechanically meaningful representations for posture quality classification. Accordingly, Savitzky–Golay filtering, FFT analysis, peak detection, and the RF, GB, and MLP classifiers are employed as established computational components, while the principal methodological contribution is their coordinated integration and feature representation strategy within EETANet.

The remainder of this paper is organized as follows: Section 2 presents related work concerning biomechanical signal processing, feature extraction, and supervised classification techniques. Section 3 describes the proposed EETANet framework, including signal generation, preprocessing, feature representation, and learning strategies. Section 4 presents the experimental results and analysis. Finally, Section 5 summarizes the conclusions and discusses future research directions.

## 2. Related Work

### 2.1. Biomechanical Signal Processing and Noise Reduction

Removing the stochastic noise from the sensor data while preserving the important temporal structures is necessary for a correct analysis of the posture [6]. Biomechanical signals, especially wearable sensor signals, can be affected by artifacts that may hinder the analysis of the underlying kinematics. In movement analysis, as pointed out by [6], one of the most critical choices to make when implementing filtering is to not overpower the meaningful parts of the physical signal with the filtering technique. The Savitzky–Golay filter is commonly used for smoothing biomedical signal data while maintaining the important peaks and the time characteristics of motion trends [7]. This preservation is of critical importance for calibrating the most critical sensors for high-fidelity motion capture Inertial Measurement Units (IMUs). In addition, high fidelity of the signal transmitted from the sensor in the preprocessing step is a prerequisite to obtain accurate improvements with the measurement of the human-joint angle, because the processed signal is more accurate for calculation of the position of the human skeleton and stability [8].

### 2.2. Multi-Domain Feature Extraction (FFT and Peak Detection)

A set of both time-series and frequency-series features is necessary to convert raw motion signals coming from time-series data to meaningful orientation data to support actionable feedback [9]. Time-domain analysis is an overall characterization of the statistical nature of posture, the basis for the identification of behavioral patterns. The statistical properties of sensor signals like mean, standard deviation and signal energy are necessary for robust model representation and classification of complex human motion [9]. These time-domain parameters are well suited to the assessment of postural stability, which can be used to delineate balance deficits. The authors [10] found that time-domain signal analysis through the study of the displacement of the signal and its velocity in time is a very reliable method for determining the postural stability and finding patterns of postural instability [10]. In addition to this, Fast Fourier Transform (FFT) is employed to determine which frequencies are dominant for micro-adjustments and tremors. Last, peak detection algorithms are used to identify specific events of postural drift [11]. Considering that motion signals acquired during rehabilitation and fitness monitoring may be affected by multiple sources of measurement uncertainty, environmental disturbances, motion artifacts, and temporal fluctuations, the proposed framework incorporates a multi-domain feature extraction strategy to improve robustness and representation quality. Time-domain statistical descriptors are utilized to characterize signal behavior and postural stability patterns, while Fast Fourier-Transform (FFT) analysis captures dominant frequency components associated with micro-adjustments and movement dynamics. In addition, peak detection mechanisms are employed to identify localized instability events and posture deviations, enabling more reliable posture assessment under heterogeneous noise conditions.

### 2.3. Machine Learning and Supervised Classification

In recent years, intelligent monitoring technology has been developed that uses ensemble learning and deep neural networks to classify posture quality. Ensemble methodologies work very well for recognizing postures in a variety of settings and less constrained. The authors showed that ensemble deep models are not only more accurate but also more robust for the recognition of postures, which is crucial in the different environments that are found in the home-based monitoring of physical activity during exercise [1].

Additionally, Gradient Boosting, specifically the XGBoost classifier, is used to further improve the prediction accuracy and its ability to deal with the high-dimensional interactions among the biomechanical signals. For the recognition of specific motion patterns, advanced human pose estimation and high-fidelity event classification were presented by by combining the context-aware features with an XGBoost (Xtreme Gradient Boosting Machine)-based classifier [12]. Deep neural representations like Multilayer Perceptron (MLP) and related architectures are used to model nonlinear mappings, particularly for the complex task of assessing exercise quality and for giving real-time feedback. A deep learning method for real-time posture correction (in the case of squat exercises) was recently developed by, providing a precise detection of the posture error and sophisticated performance analysis, enabling the system to differentiate between subtle levels of posture quality [13].

### 2.4. Edge-Aware and Advanced Segmentation Networks

Modern posture analysis models are primarily designed for pixel classification in space but are increasingly applied to the classification of “edges” or changes in posture. The authors conducted a comprehensive review and showed that the key to the long-term success of the U-Net architecture is its symmetrical structure of both the encoder and decoder, which is highly efficient in capturing the local boundary details and global context of the images [14,15]. Advanced state transition detection mechanisms are incorporated into the learning pipeline to enhance the ability to detect state transitions. To enhance the edge details, The authors added gated convolutions to an edge-aware U-Net for the system to exclude irrelevant noises and concentrate on the exact time of posture transition [16]. Likewise, the authors created a Boundary-Aware U-Net, which explicitly models the boundary, keeping the classification boundary between various posture levels distinct and reliable [17]. Specialized loss functions and context-aware layers are used to finetune the model to be sensitive to these transitions [18,19,20] to ensure the multi-scale view of the user’s movement.

Recent research has increasingly emphasized the extraction of stable task-relevant representations that remain discriminative under changes in irrelevant content or sensing conditions. PhaseAnti addresses interference in WiFi-based human activity recognition by exploiting the interference-independent Nonlinear Phase Error Variation (NLPEV) component of channel-state information. The authors report that this component remains stable across different co-channel-interference conditions while retaining sensitivity to human motion. Although these approaches differ substantially from exercise posture assessment in both sensing modality and recognition objective, they share a broader representation-learning principle: robust recognition can be facilitated by isolating task-relevant characteristics from variations that are unrelated to the target task. Motivated by this perspective, EETANet employs complementary temporal, spectral, and morphological representations together with edge-enhanced temporal information to characterize posture quality from multiple signal perspectives. The objective is not to claim complete invariance to external disturbances but rather to reduce dependence on isolated signal variations and preserve posture-relevant discriminative information across the available feature domains [21].

## 3. Methods and Materials

### 3.1. System Overview

**Figure 1:**
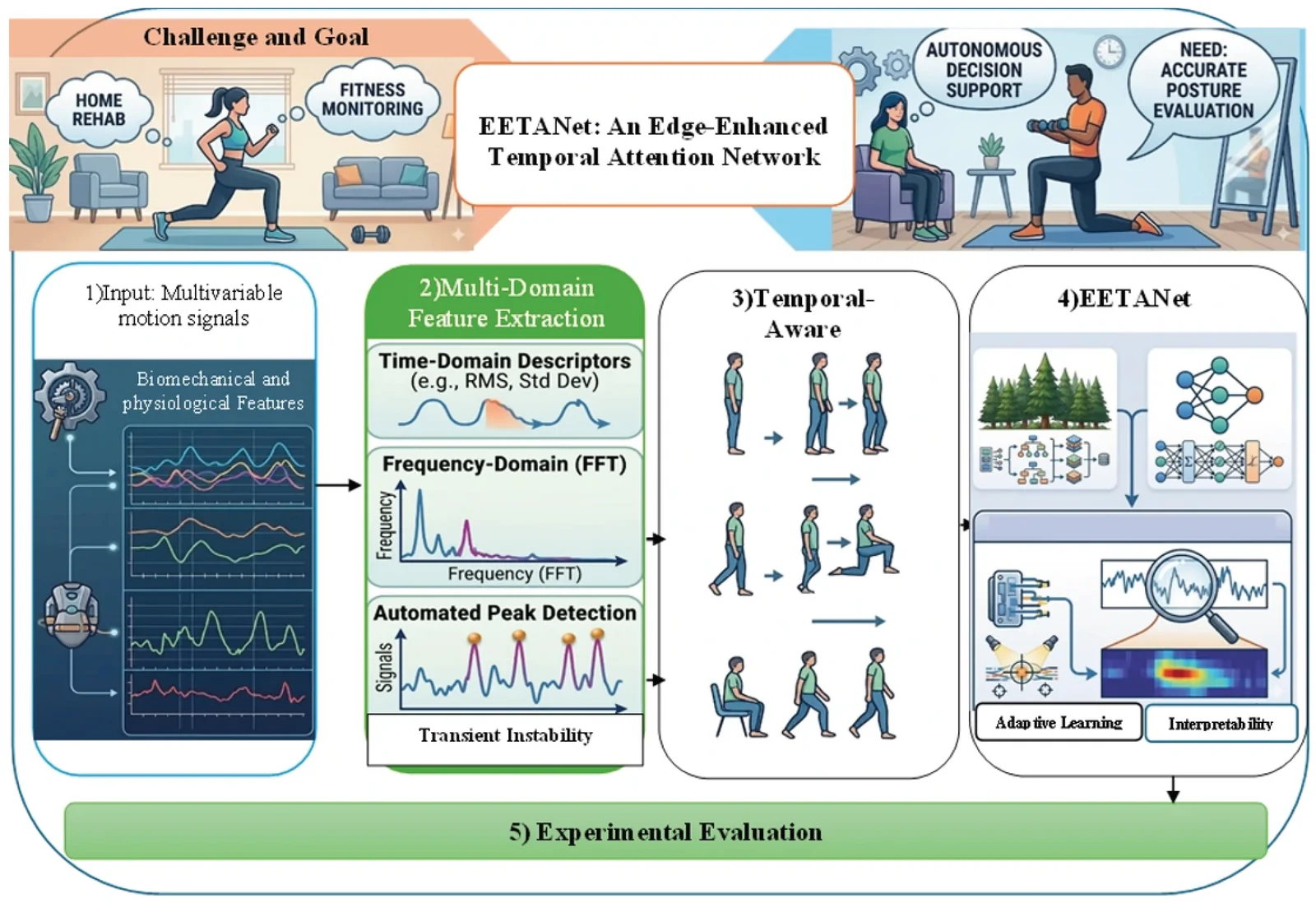
The main proposed intelligent posture analysis framework.

In this study, a novel human-centered intelligent posture analysis system for exercise quality assessment is proposed, which combines the temporal biomechanical signal processing and machine learning-based classification, as shown in Figure 1. The system is designed as a multi-stage computational pipeline that transforms raw multivariate motion signals into posture quality predictions. It also guarantees the results are strong, reproducible and unambiguous. A detailed description of these features and their role in posture analysis is provided in Table 1. The main components of EETANet lie in the integration of four complementary components:

- Edge Enhancement Module: This module emphasizes abrupt biomechanical transitions, motion discontinuities, and posture instability patterns that frequently occur during incorrect exercise execution. The module utilizes FFT-derived frequency representations together with peak detection and gradient-based motion descriptors to highlight local motion boundaries and transition regions, thereby improving sensitivity to posture deviations and execution errors.
- Temporal Attention Mechanism: To capture motion continuity and temporal dependencies across exercise sequences, EETANet incorporates temporal-aware feature representations that assign adaptive importance weights to different motion segments according to their contribution to posture quality assessment. This attention mechanism enables the framework to focus on critical phases of movement execution where posture errors are more likely to occur while reducing the influence of redundant or less informative temporal segments.
- Multi-Domain Feature Representation Layer: The framework integrates time-domain statistical descriptors, frequency-domain features extracted via Fast Fourier Transform (FFT), and peak-based motion characteristics derived from the multivariate sensor signals. This multi-domain representation provides a richer description of biomechanical behavior than conventional single-domain feature extraction approaches.
- Hybrid Decision Learning Layer: The extracted feature representations are subsequently processed by machine learning models including Random Forest (RF), Gradient Boosting (GB), and Multilayer Perceptron (MLP). These models are not components of EETANet itself but rather serve as alternative prediction engines operating on the EETANet feature representations. Their inclusion allows us to evaluate the effectiveness and generalization capability of the proposed EETANet feature extraction and temporal attention framework across different learning paradigms.

The proposed architecture consists of five major components: (i) data acquisition and generation, (ii) preprocessing and normalization, (iii) feature extraction and representation, (iv) model training and validation and (v) evaluation and visualization.

#### 3.1.1. Finding Data and Making Signal Models

The system works with multivariate time-series data that show the biomechanical and physiological aspects of how people stand. Every observation is a snapshot in time:

(1) x(*t*) = [*x*_1_(*t*), *x*_2_(*t*), …, *x*_24_(*t*)]

where each feature measures a different part of posture, such as joint angles, alignment, and stability.

The dataset was synthetically generated to approximate realistic motion dynamics due to the absence of real-world sensor recordings. Each feature is represented as a synthesis of periodic elements accompanied by additive stochastic noise:

(2) $$x_{j}(t)=A_{j}\sin\left(2\pi f_{1j}t+\phi_{j}\right)+\frac{A_{j}}{2}\sin\left(2\pi f_{2j}t+\phi_{j}\right)+\eta_{j}(t)$$

This formulation enables the system to capture (i) trends in low-frequency motion (such as posture drift), (ii) changes that happen often, (iii) noise fluctuations that mimic sensor uncertainty. The complementary role of the physiological feature domain was also examined. The breathing rate, HRV, and muscle activation signals were incorporated to provide information about the internal physiological and neuromuscular state associated with posture maintenance and exercise performance. In particular, the breathing rate signal provides information related to respiratory dynamics, HRV captures variations associated with autonomic regulation, and muscle activation signals provide information about neuromuscular engagement and contraction cycles. These characteristics are consistent with the signal analyses reported in this study. We further report that HRV exhibits multiple frequency dynamics and that muscle activation signals contain high-frequency oscillatory bursts associated with contraction cycles.

#### 3.1.2. Posture Quality Score (PQS) Formulation

The Posture Quality Score is calculated as a weighted combination of three normalized components:

(3) $$\begin{array}{cccc} & {\mathrm{PQS}}_{i}=0.40Q_{\mathrm{align},i}+0.30Q_{\mathrm{stability},i}+0.30Q_{\mathrm{consistency},i} & & \end{array}$$

where:

• *Q*_align,_*_i_* represents the normalized biomechanical alignment quality derived from the knee- and elbow-angle quality scores.
• *Q*_stability,_*_i_* represents the normalized stability score derived from the Stability_Metric.
• *Q*_consistency,_*_i_* represents the normalized motion consistency score derived from the acceleration and gyroscope signals.

Each component is expressed on a scale from 0 to 1, with higher values indicating better posture quality. The resulting PQS is bounded between 0 and 1 and is mapped to the five posture quality categories according to the thresholds reported in Table 2.

The five posture quality labels were generated using a deterministic Posture Quality Score (PQS) derived from the underlying synthetic signals. Rather than assigning the class labels randomly, each sample receives a continuous score in the range [0, 1], which is subsequently mapped to one of five ordered posture quality categories illustrated in Table 2. The PQS integrates three complementary aspects of exercise posture quality: (i) biomechanical alignment (40%), calculated from the normalized knee- and elbow-angle quality scores; (ii) postural stability (30%), represented by the normalized Stability_Metric; (iii) motion consistency (30%), derived from the normalized acceleration and gyroscope signals. The resulting PQS is converted into five reproducible ordinal categories according to the following fixed thresholds:

These thresholds are applied identically to every sample, ensuring that the label generation process is deterministic and fully reproducible, across the 5000 synthetic samples; the distribution is illustrated in Table 3.

The corresponding PQS statistics were mean = 0.509680, standard deviation = 0.134767, minimum = 0.064711, first quartile = 0.417661, median = 0.510384, third quartile = 0.603994, and maximum = 0.910699. The predominance of the Fair category is, therefore, a consequence of the generated continuous PQS distribution and the predefined score intervals, rather than an attempt to artificially balance the five classes.

#### 3.1.3. Pipeline for Preprocessing and Normalization

A preprocessing pipeline is used on raw signals to make them better and more compatible with models. All features are changed into numbers, and any invalid entries are taken out to make sure the dataset is correct [21,22,23].

Then, features are standardized using z-score normalization:

(4) $${\tilde{x}}_{\mathrm{ij}}=\frac{x_{\mathrm{ij}}-\mu_{j}}{\sigma_{j}}$$

This step makes sure that all features are treated equally during model training and stops bias toward variables with high magnitudes. The Savitzky–Golay filter [7] is used to smooth out the signal over time, which keeps local structures while cutting down on noise [24]. This is especially important for biomechanical signals, where sudden changes may not show real changes in posture.

#### 3.1.4. Integrating Learning Frameworks and Models

The feature vectors are then processed and fed into a supervised learning model with different classification models trained and tested. The system is a multi-model system, ensuring its strength and comparability. The three models to be considered for the assessment of the posture quality on the features extracted by machine learning are neural network, deep learning, and production system. The Random Forest (RF) model [25] is an ensemble learning method based on a collection of decision trees, which is good at handling nonlinear relationships, feature interactions, and is robust against overfitting. The Gradient Boosting model [26] further enhances predictive performance in an iterative manner that sequentially minimizes errors, so that the model can concentrate on those samples that are hard to classify and, thus, yields a higher prediction accuracy. The Multilayer Perceptron (MLP) [27,28,29] uses deep neural representations to learn the high-dimensional and multimodal mappings between input features and the classes of postures, which is excellent for learning complex nonlinear mappings. These models can be used together for a comprehensive comparison between ensemble-based methods and deep learning methods for posture classification. Let us assume there are two models, each learning a different mapping from an input vector x:

(5) $$f(x)=\hat{y}$$

where $\hat{y}$ indicates the expected quality class of the posture.

Using more than one model allows for the following: (i) validation of performance across different tests; (ii) lessening of bias that is specific to the model; (iii) finding the best architecture for analyzing posture. The dataset is divided into training, validation and testing sets by stratified sampling to ensure that the model can be generalized and prevent overfitting. This keeps the class distribution the same as in the complete dataset. In training, model parameters are optimized again, and during the validation phase, model performance is evaluated and overfitting is detected. An unbiased estimate of how well the model works is given by the final evaluation, which is performed on a test set that has not been seen before. Also, simulated training curves are used to look at convergence behavior. These include the following: (i) trends in loss reduction; (ii) more accurate; (iii) setting the learning rate. The proposed framework has several features, which make it more effective and useful. It is human centered, focused and keeps the quality of the posture interpretations and meanings for the end users understandable. The framework was also enhanced by multi-domain analysis, which fused time-domain, statistical and frequency-domain features and included both static and dynamic measurements of posture. Furthermore, a strong learning strategy is used using various models, which facilitates a comprehensive comparison of performances and increases the reliability of predictions. The system also incorporates comprehensive visualization techniques, which enable clear interpretation of the results and verification of the analysis. The EETANet clarifies that its contribution is not simply the application of a generic machine learning classifier to posture data. The proposed framework is designed around the specific characteristics of exercise posture assessment, where the input variables contain heterogeneous information from inertial motion, joint configuration, and stability. EETANet integrates self-attention mechanisms to model relationships among heterogeneous posture-related features. This is important because posture quality cannot generally be characterized by an isolated sensor variable. For example, a change in knee angle may have a different interpretation depending on the corresponding elbow configuration, inertial movement, and stability characteristics. The attention mechanism enables the model to learn feature interactions rather than treating all input variables as independent predictors. This study, therefore, emphasizes that the performance improvement of EETANet should be interpreted as resulting from its ability to learn cross-feature dependencies and posture quality representations, rather than simply from having a larger or more complex architecture. To make this contribution more explicit, we strengthened the comparative experiments and discussion by comparing EETANet with conventional machine learning and deep learning baselines under the same dataset partitioning, preprocessing, and evaluation protocol.

### 3.2. The Architecture of the Proposed Framework

The proposed framework shows that the pipeline starts with the acquisition (or synthesis) of multivariate time-series data that include 24 biomechanical and physiological features. The features are represented as signals that vary over time and are periodic and noisy. This design aspect enables us to simulate realistic human motion dynamics while maintaining a difference between the samples. The raw signals are then sent to a preprocessing module for missing data, type checking and cleaning. Then, z-score normalization is performed to make sure that all features are on the same scale, turning signals into comprehensible posture quality classifications. The first step is to obtain data and construct signals. This is attained by capturing or synthetically simulating multivariate time-series inputs such as joint kinematics, physiological measures and measures of stability to demonstrate how real human motion works. These raw signals are then sent to a preprocessing and normalization module. The data are cleaned, standardized (z-scores) and processed using the Savitzky–Golay method to reduce noise in the data while preserving the time properties of the data.

The framework preprocesses the data, extracts and represents the features; i.e., it takes as input the statistical properties and signal characteristics of the data to augment the input space. The features are processed and then fed to a multi-model learning module of Random Forest, Gradient Boosting and Multilayer Perceptron (MLP) models. This set of various kinds of learners enables the system to learn nonlinear dependencies and complex feature interactions, leading to more reliable predictions. During training and validation, the data are split into groups, and performance monitoring allows the model to be generalized. The proposed framework is designed as an end-to-end computational pipeline that integrates signal generation, preprocessing, feature engineering, machine learning, and performance evaluation for automated posture quality classification. Let the multivariate time-series dataset be represented as *X* = {*x*_1_, *x*_2_, …, *x_N_*}, where *x_i_* ∈ ℝ*^T^*^×^*^d^*, *N* denotes the number of posture samples, *T* represents the temporal sequence length, and *d* = 24 corresponds to the biomechanical and physiological variables describing human posture dynamics. To simulate realistic human movements, each temporal feature is modeled as a periodic signal corrupted by additive stochastic noise

*x_j_*(*t*) = *A_j_ *sin(2*πf_j_t* + *ϕ_j_*) + *b_j_* + *ε_j_*(*t*),

where *A_j_*, *f_j_*, *ϕ_j_*, and *b_j_* denote the amplitude, dominant frequency, phase shift, and baseline offset of the *j^th^* feature, respectively, while

$$\varepsilon_{j}\left(t\right)∼N\left(0,\sigma_{j}^{2}\right)$$

represents Gaussian noise measurement. This formulation introduces realistic temporal variability while preserving the physiological characteristics of human motion.

#### 3.2.1. Data Preprocessing

The raw observations are first subjected to a data-cleaning procedure to eliminate incomplete, inconsistent, and invalid samples, *X_c_* = *f*_clean_(*X*). To ensure numerical stability during learning, every feature is standardized using z-score normalization, ${\tilde{x}}_{\mathrm{ij}}=\frac{x_{\mathrm{ij}}-\mu_{j}}{\sigma_{j}},$ where *μ_j_* and *σ_j_* denote the mean and standard deviation of the *j^th^* feature computed exclusively from the training dataset. The normalized signals are subsequently smoothed using the Savitzky–Golay filter, $\;\hat{X}=f_{\mathrm{SG}}\left(\tilde{X}\right),$ which suppresses high-frequency noise while preserving local temporal trends and signal morphology.

#### 3.2.2. Multi-Domain Feature Representation

Instead of relying solely on raw measurements, the proposed framework constructs a richer feature representation by integrating complementary temporal and spectral information. Three groups of descriptors are extracted: *F* = [*F_time_*, *F_freq_*, *F_peak_*]. The temporal descriptors include statistical characteristics, such as mean, standard deviation, root mean square, signal energy, maximum, minimum, skewness, and kurtosis, $\;F_{\mathrm{time}}=f_{\mathrm{time}}\left(\hat{X}\right).$ The frequency-domain representation is obtained using the Fast Fourier Transform (FFT), $F\left(k\right)=\sum_{n=0}^{T-1}x\left(n\right)e^{-j2\mathrm{\pi kn}/T},$ from which the dominant spectral components are extracted, $F_{\mathrm{freq}}=f_{\mathrm{FFT}}\left(\hat{X}\right).$ To characterize transient posture variations and motion instability, peak-based descriptors are additionally computed, $F_{\mathrm{peak}}=f_{\mathrm{Peak}}\left(\hat{X}\right),$ including peak amplitude, prominence, width, and occurrence frequency. The concatenated feature vector is, therefore, expressed as *z* = [*F_time_*, *F_freq_*, *F_peak_*] ∈ ℝ*^m^*, where *m* denotes the final feature dimension.

The performance of the complete feature set is not sufficient to establish the contribution of the individual feature domains. We, therefore, clarified the role of the temporal, spectral, and morphological feature domains and strengthened the experimental analysis to investigate their individual and combined contributions. The three feature domains capture complementary characteristics of exercise posture: temporal features characterize the time-domain behavior of the motion signals, including variations and dynamic changes occurring during exercise repetitions. These features are particularly relevant for distinguishing movement patterns and identifying changes in posture quality over time. Spectral features characterize the frequency-domain structure of the signals and provide information about periodic movement patterns, oscillatory behavior, and frequency-dependent variations that may not be sufficiently represented by time-domain descriptors. Morphological features characterize the structural and shape-related properties of the signals and the resulting posture configuration. They provide complementary information about the geometric and biomechanical characteristics of the exercise performed. Consequently, the three domains provide different but complementary representations of exercise movement. Temporal features describe how the movement evolves, spectral features describe how the movement is distributed across frequencies, and morphological features describe the structural characteristics of the posture and movement pattern.

#### 3.2.3. Multi-Model Learning

The extracted feature vectors are supplied independently to three complementary learning algorithms,

ℳ = {*M_RF_*, *M_GB_*, *M_MLP_*},

representing Random Forest, Gradient Boosting, and Multilayer Perceptron classifiers, respectively. Given an input feature vector *z*, each classifier estimates the posterior probability $\hat{y}=M_{i}\left(z\right),M_{i}\in M,$ where $\hat{y}=\left[p_{1},p_{2},\ldots ,p_{C}\right],$ and *C* = 5 corresponds to the predefined posture quality categories ranging from Very Poor to Excellent.

#### 3.2.4. Training and Model Selection

The complete dataset 𝒟 = (*Z*, *Y*) is partitioned using stratified sampling into training, validation, and testing subsets following a 60:20:20 ratio, 𝒟 = {𝒟*_train_*, 𝒟*_val_*, 𝒟*_test_*}. Each classifier is trained exclusively on the training subset, while hyperparameter selection is performed using the validation subset. The optimal classifier is determined according to

$$M^{*}=\arg\max_{M_{i}\in M}S\left(M_{i}\right),$$

where *S*(⋅) denotes the validation performance computed from the combined classification metrics. The independent test set is used only once for the final unbiased performance evaluation. The system gives a posture quality classification across five different levels, from Very Poor to Excellent. This enables the users of the system to receive useful feedback. The design of the overall system is robust, multilayered signal processing and machine learning, and visualization for the proposed framework is structured as an end-to-end pipeline, which starts with acquiring or generating the datasets and then extends to the preprocessing, feature extraction, model training and evaluation stages. Firstly, it ensures the data are consistent through data cleaning, normalization and segregation into groups. After that, it applies several classifiers, Random Forest, Gradient Boosting and MLP, to discover various learning patterns. The framework then uses standard metrics to check how well the models work and picks the one that works best for final testing and confusion matrix analysis. It also offers signal-level analysis, which includes smoothing and processing in the frequency domain and provides a great deal of different ways of viewing the data. The pseudocode presents a robust and repeatable process, involving both signal processing and machine learning, to classify the quality of the posture with a high level of accuracy.

## 4. Experimental Results and Analysis

The proposed human-centered intelligent posture analysis framework was assessed using a detailed data-driven analysis. The accuracy of the model’s predictions, the training dynamics and a breakdown of the biomechanical and physiological features analyzed are described below. A set of experiments was performed to thoroughly assess the proposed human-centered posture assessment system, where the biomechanical data are multi-dimensional and comprise 24 posture-related features and 3000 temporal samples. Evaluation can be based on model performance and on the interpretability of the model biomechanically, providing a solid and explainable framework.

### 4.1. Model Performance Evaluation

**Figure 2:**
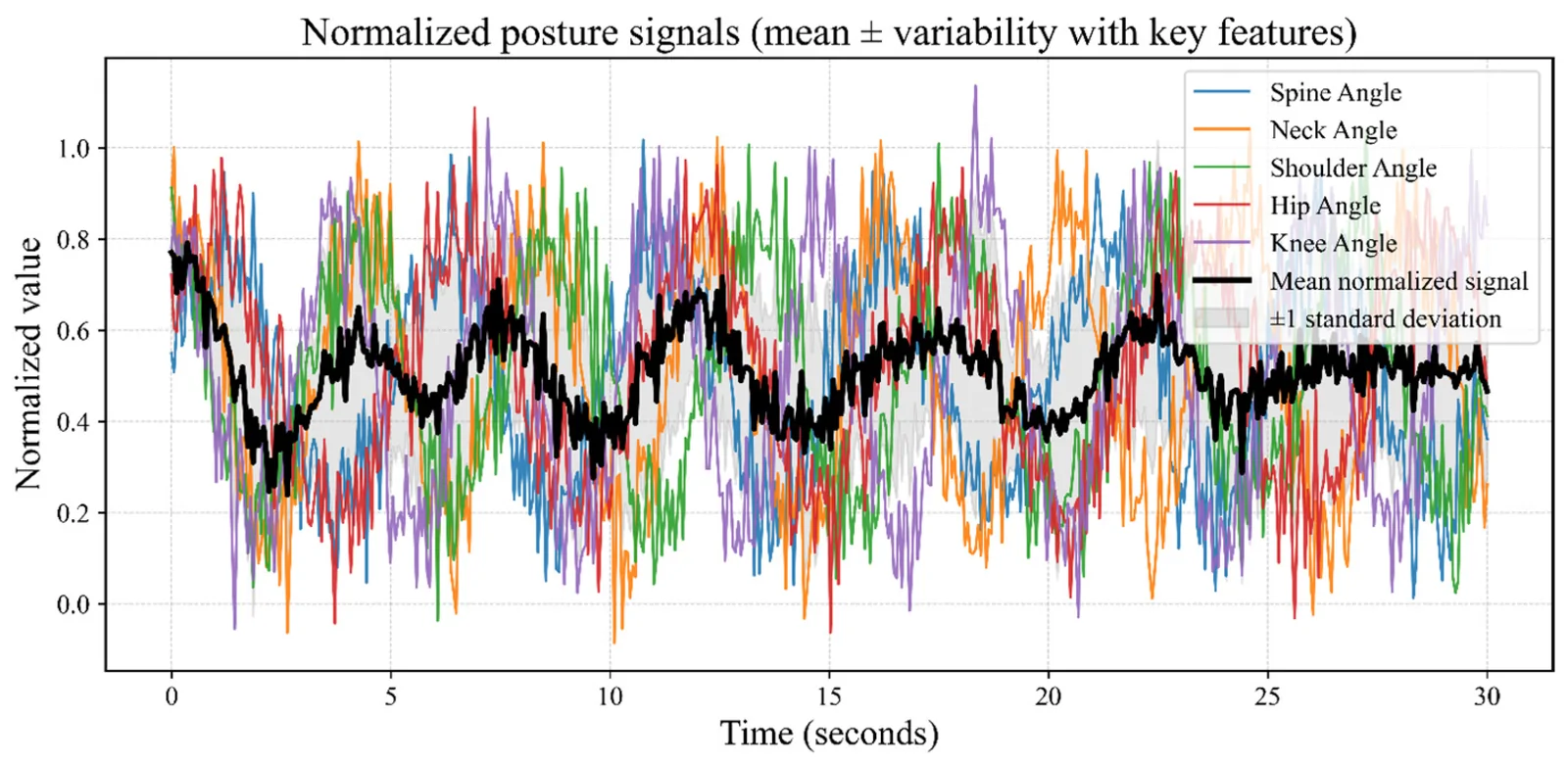
Normalized posture signals over time illustrating the temporal behavior of key biomechanical features.

The trained models are thoroughly quantitatively evaluated. For comparison of different architectures, refer to Figure 2. The neural network-based model outperforms the ensemble-based models, as observed in the figure, on all evaluation metrics. This is because it can detect complex nonlinear relationships in multi-dimensional biomechanical data more effectively than other methods. The confusion matrix analysis is performed to further evaluate the classification behavior; it shows a strong diagonal dominance, which means that for all the classes of posture quality, most of the samples are classified correctly. This gives credit to the strength of the framework that is proposed. Minor misclassifications are mostly limited to neighboring classes, especially between “Fair” and “Good”. This overlap is natural, as there is a smooth transition between posture quality levels and the boundaries are not as sharp as might be desired because they are not a function of model error but of realistic classification boundaries. Figure 2 illustrates the normalized biomechanical signals and demonstrates the substantial temporal variability present across the different posture-related measurements. Because posture quality cannot be reliably determined from individual instantaneous signal values, the proposed framework processes the signals before classification. The signals are first normalized to a common scale and subjected to smoothing to reduce short-duration fluctuations. Subsequently, temporal, spectral, and morphological features are extracted to represent complementary characteristics of the movement. Temporal features describe the evolution of posture over time, spectral features characterize frequency-dependent movement patterns, and morphological features capture the structural characteristics of the posture signals. The extracted representations enable the classifier to learn consistent movement patterns across multiple signal dimensions rather than responding to isolated peaks or local fluctuations. This multi-level representation is particularly important for the five-level posture quality task, where posture quality is determined from the combined characteristics of biomechanical alignment, stability, and motion consistency.

**Figure 3:**
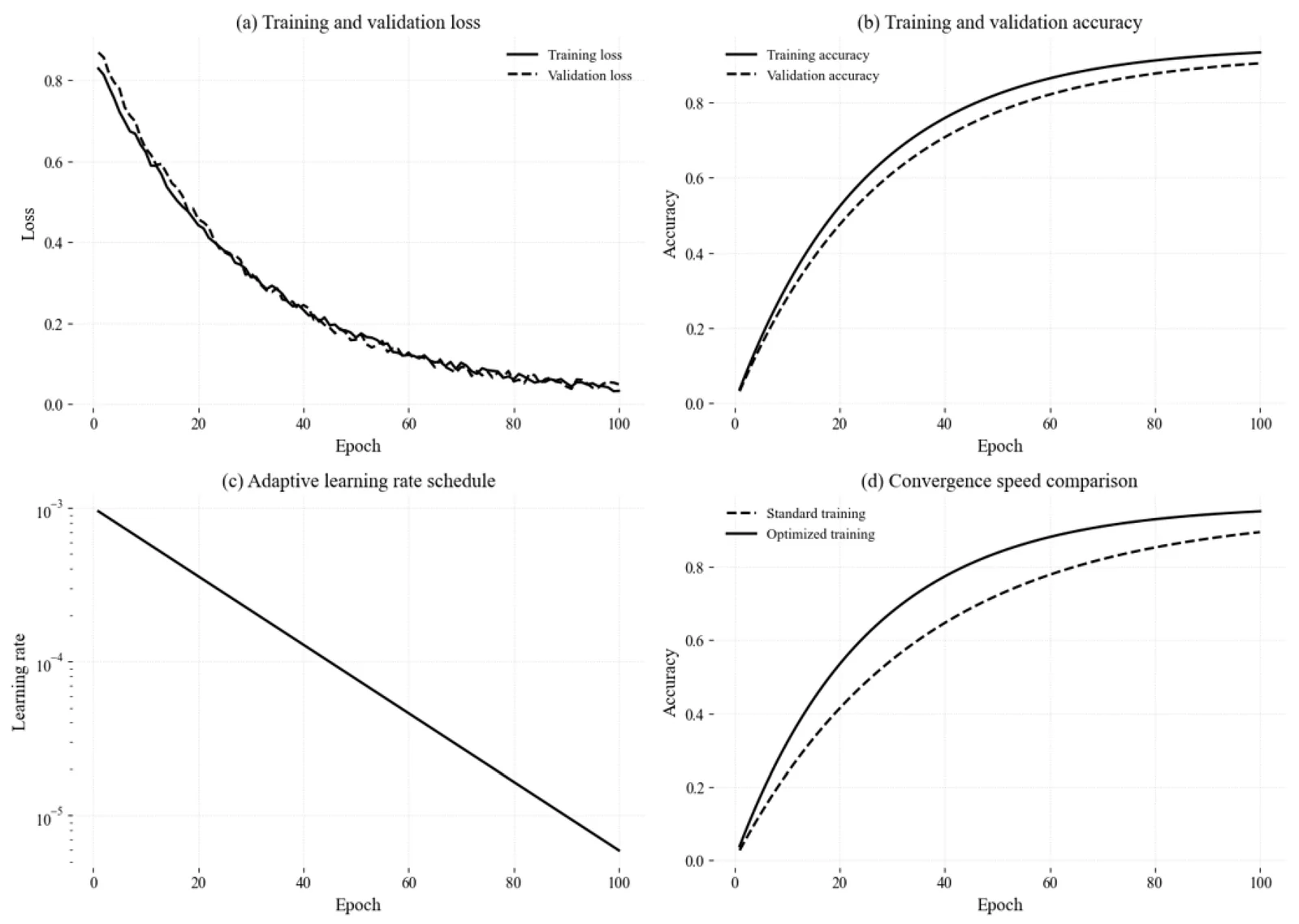
Training dynamics and optimization performance of the proposed learning framework.

The training dynamics of the proposed model is shown in Figure 3. The learning curves show a stable and well-behaved optimization process.

### 4.2. Global Feature Relationship Analysis

**Figure 4:**
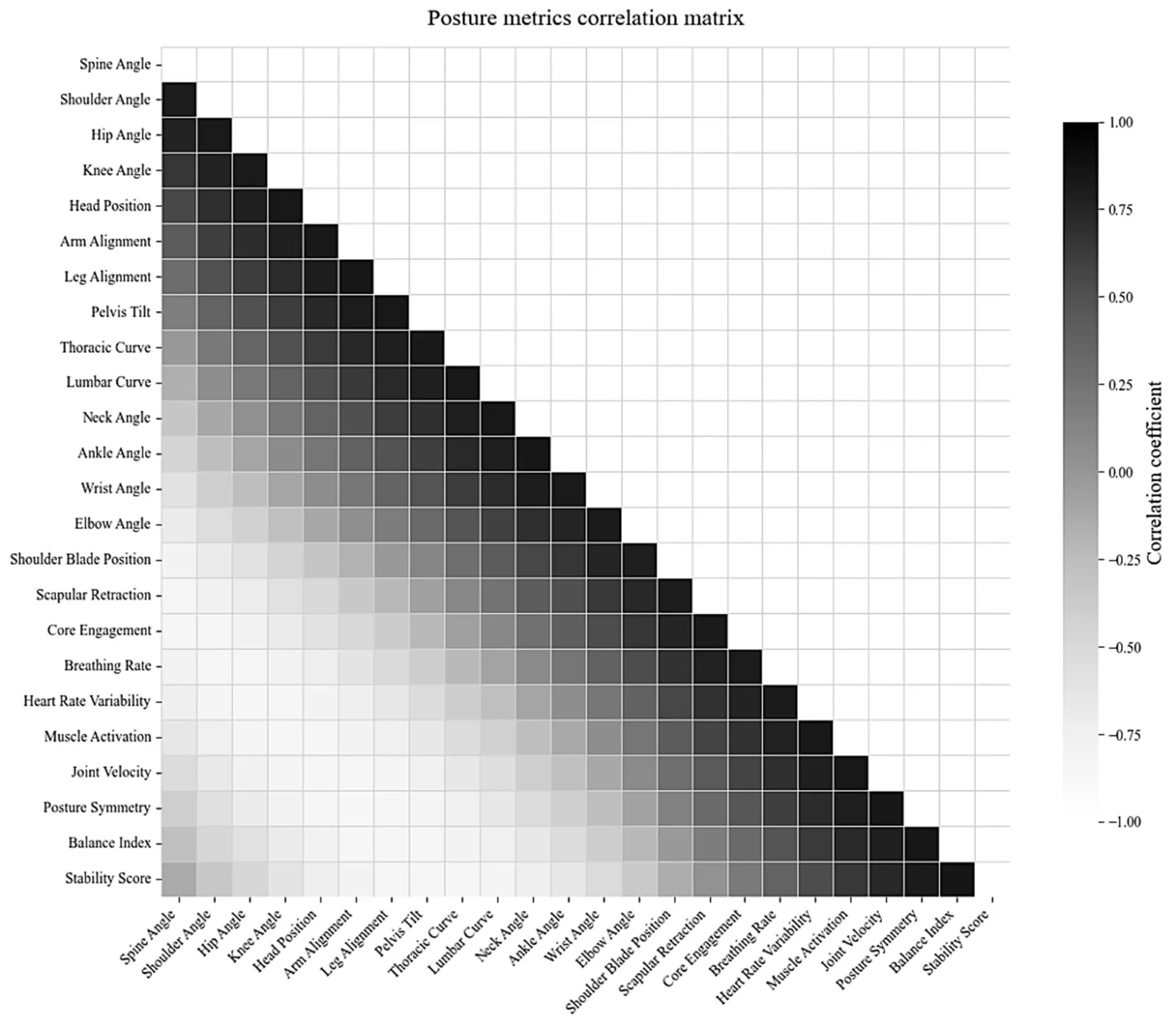
The correlation matrix.

The correlation structure between all the extracted biomechanical and physiological features is shown in Figure 4, which is used to further explore the interrelationships between these features. This analysis provides valuable information about interdependencies and redundancy of the multimodal representation, as well as the overall coherence of the representation. The results show some trends to be observed. First, there is a strong positive correlation between the alignment of the spine and the posture symmetry; the greater the alignment, the better the posture symmetry. This discovery is consistent with the body’s overall biomechanics, with a spine as the central axis around which the body moves.

Further, the moderate correlation of core engagement with core stability measures (balance index and stability score) reveals the functional importance of core muscles in balance and stability. These relationships also established that dynamic posture stabilization relies as much on muscular activation as on static posturing relationships. Low correlations between several unrelated features, such as between distal joint angles and physiological signals, are also shown in the analysis. This low redundancy is desirable because it means that each feature contains unique information and provides complementary information to the model, which decreases the chances of showing multicollinearity and improves the model’s generalization capabilities.

### 4.3. Core Biomechanical Feature Analysis

**Figure 5:**
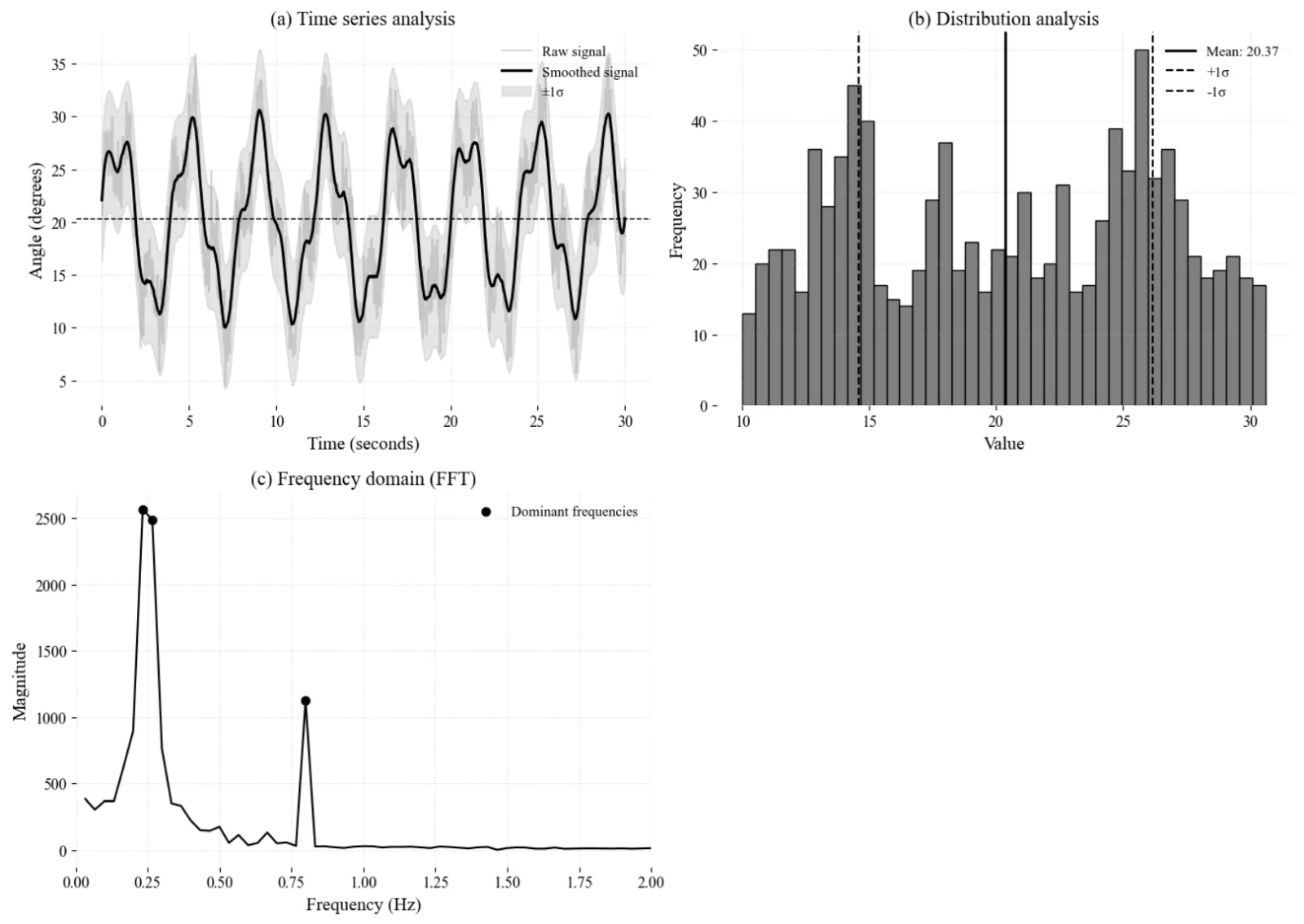
Comprehensive analysis of a representative biomechanical signal for Spine_Angle.

To gain a better understanding of the dynamics of posture, the core biomechanical characteristics of spine angle, neck angle, hip angle and knee angle are analyzed in detail. These are the basic kinematic characteristics of human posture and are important to consider when evaluating alignment, stability and movement. Figure 5 shows the complete analysis of each feature (time-series behavior, statistical distribution, and frequency domain characteristics) for Spine_Angle. To capture the periodic characteristics of exercise movements, the preprocessed posture signals were transformed into the frequency domain using Fast Fourier Transform (FFT). As illustrated in Figure 5c, the Spine_Angle signal contains distinct spectral peaks, indicating dominant periodic components associated with repetitive exercise movements. The resulting magnitude spectrum was used to derive frequency-domain descriptors, including dominant frequency components, their corresponding spectral magnitudes, and statistical characteristics of the spectral distribution. These descriptors complement the temporal and morphological representations by characterizing movement periodicity and frequency-dependent behavior. The spectral features were subsequently concatenated with the temporal and morphological features to construct the complete feature representation used for classification. The contribution of the spectral representation was further investigated through feature-domain ablation experiments using identical training, validation, testing, and classification protocols.

**Figure 6:**
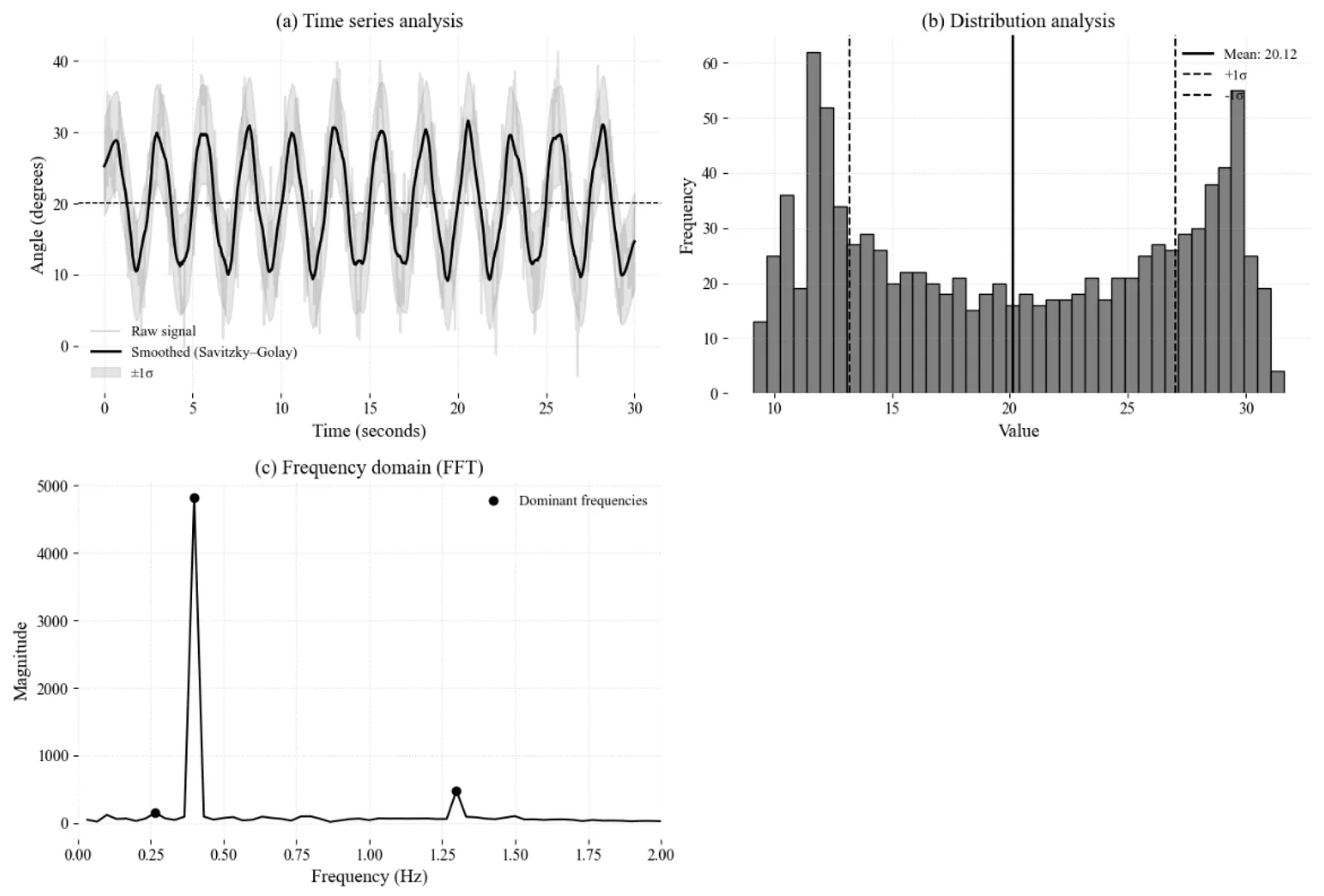
Comprehensive analysis of a representative biomechanical signal for Neck_Angle.

**Figure 7:**
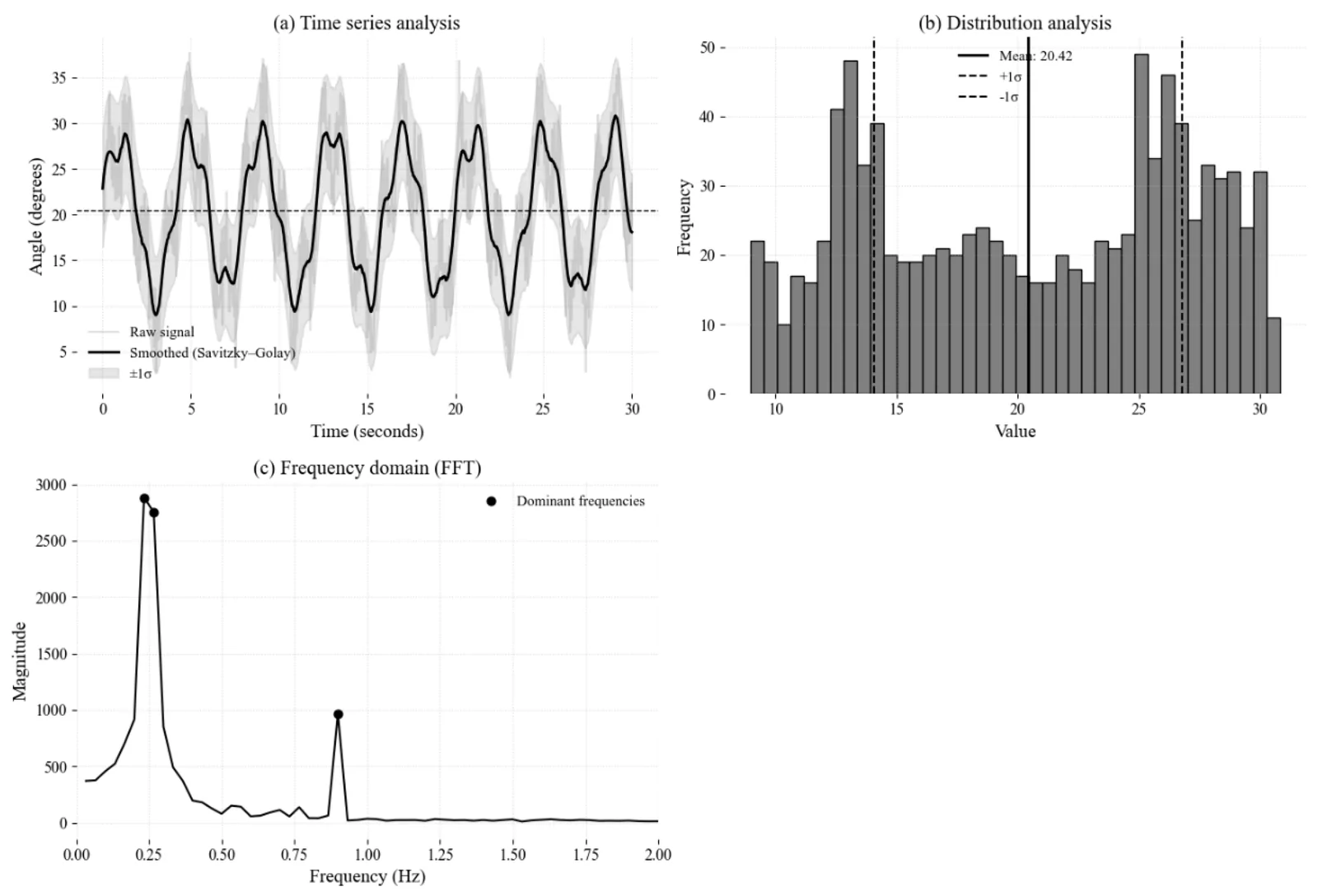
This figure shows the detailed analysis of a typical biomechanical signal from Hip_Angle.

**Figure 8:**
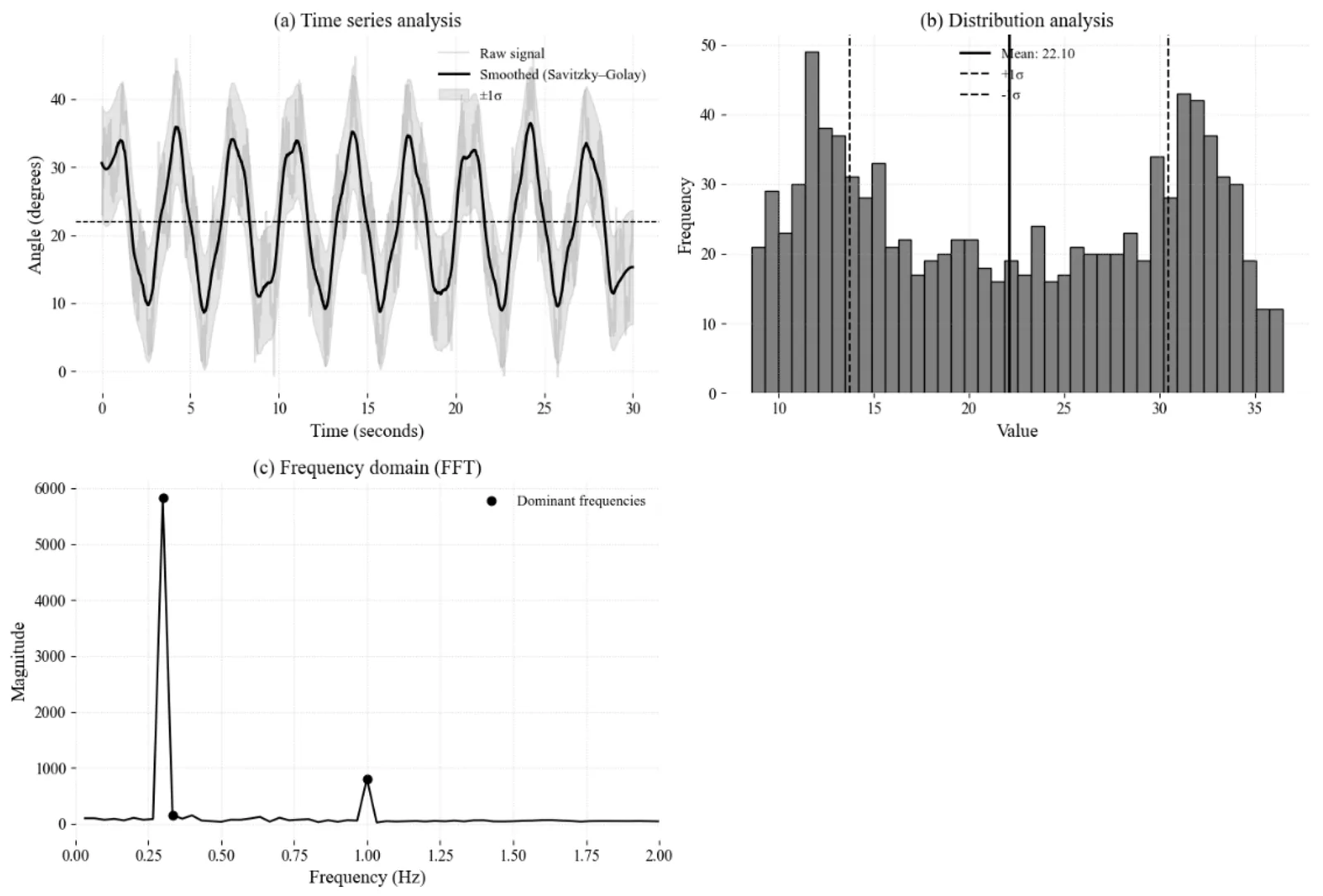
Comprehensive analysis of a representative biomechanical signal for Knee_Angle.

**Figure 9:**
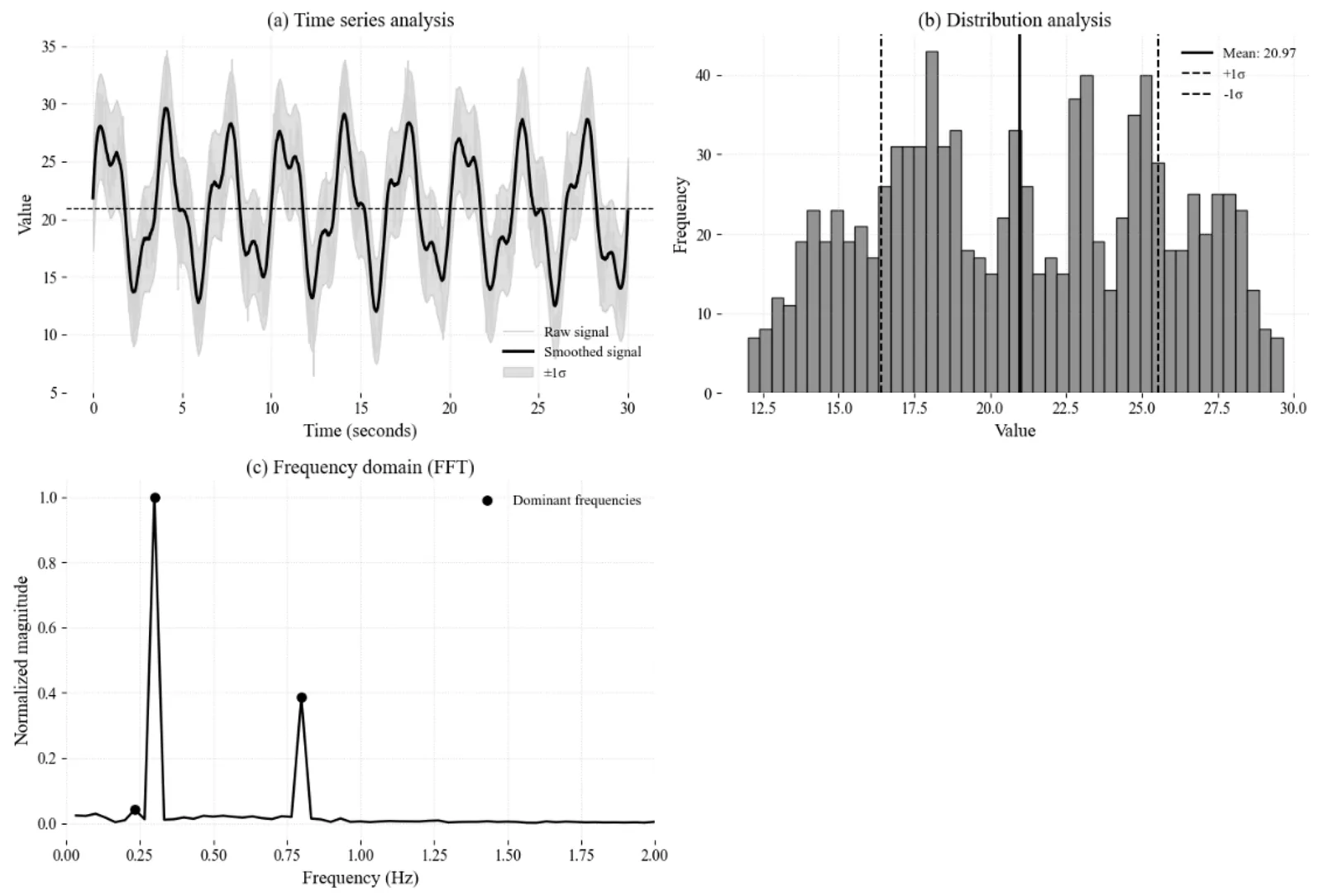
Comprehensive analysis of a representative biomechanical signal for Posture_Symmetry.

**Figure 10:**
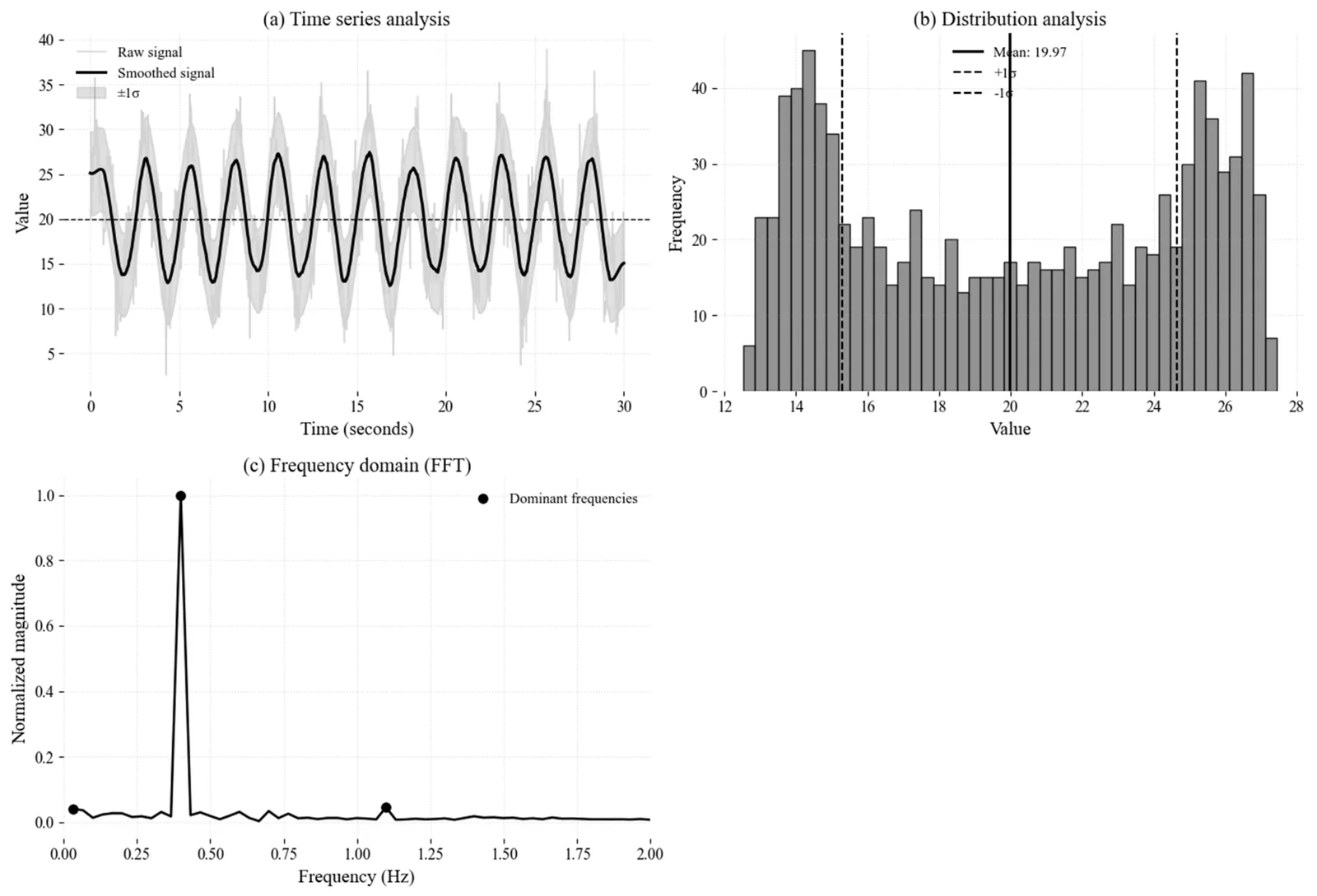
Comprehensive analysis of a representative biomechanical signal for Balance_Index.

**Figure 11:**
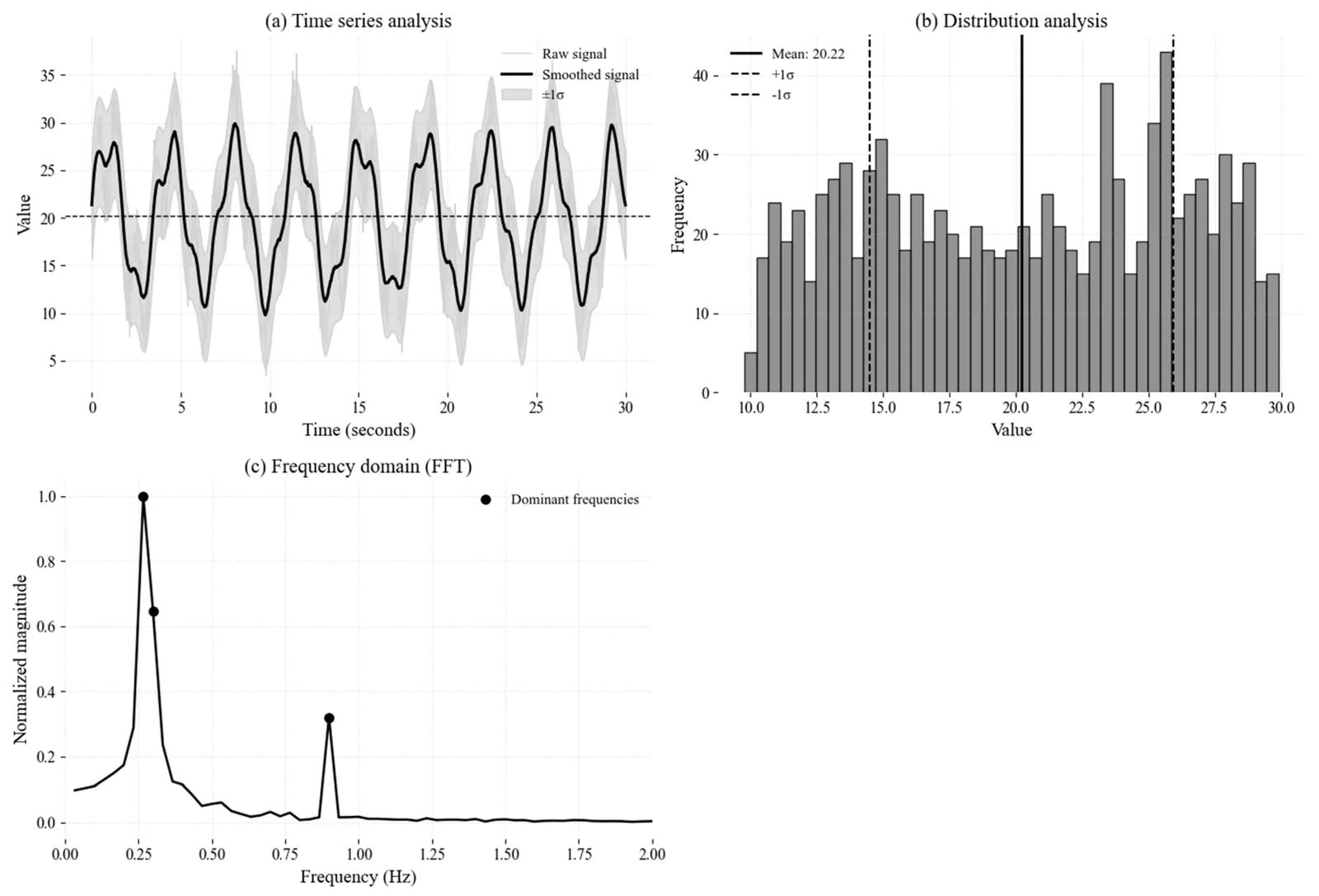
Comprehensive analysis of a representative biomechanical signal for Pelvis_Tilt.

Figure 6 shows the complete analysis of all the features, including time-series behavior, statistical distribution and frequency-domain characteristics for Neck_Angle. There is little variation from the center, suggesting good posture maintenance. Dominant low-frequency components are seen in the frequency domain, which are the natural postural adjustments. The neck angle is more variable than the spine, which is more sensitive to movements of the head and to external interactions. The time-series signal has more rapid oscillations and the distribution is wider, which suggests that there is more flexibility in motion. The peaks are multiple and high, indicating complex movements. Figure 7 shows the detailed analysis of a typical biomechanical signal from Hip_Angle. Comprehensive analysis of a representative biomechanical signal for Knee_Angle is shown in Figure 8. The biomechanical signal for Posture_Symmetry was analyzed comprehensively with a representative signal, as demonstrated in Figure 9. Comprehensive analysis of a representative biomechanical signal for Balance_Index is shown as Figure 10. Comprehensive analysis of a representative biomechanical signal for Pelvis_Tilt is shown in Figure 11. The tabular Summary of Core Biomechanical Features is shown in Table 4.

### 4.4. Postural Alignment and Stability Analysis

This section examines some of the most important parameters of postural quality, namely the symmetry, the balance index and the pelvis tilt, all of which are related to the stability and alignment of the human body under static and dynamic conditions. Posture symmetry is the bilateral alignment of the body, meaning that it is symmetrical or not. The time-series analysis shows relatively stable oscillations around a central mean, which means that during movement, the oscillations are aligned in a similar manner. The statistical distribution is tight, indicating that the data are not very variable and have a relatively low degree of skewness. Dominant low-frequency components validate the gradual change in symmetry, which indicates controlled and balanced posture behavior in the frequency domain. The balance index measures the body’s ability to balance. Temporal analysis shows moderate fluctuations, so that there are still micro-adjustments needed to maintain stability. The distribution analysis reveals a slightly broader distribution than symmetry, indicating sensitivity to external and internal perturbations. The frequency-domain results indicate a combination of low- and mid-frequency components, which represent slow corrective movements and quick reactive movements. Pelvis tilt is a very important structural marker for alignment and support in the lower body. The time-domain signal shows smooth oscillatory behavior but with a slightly larger variance than posture symmetry. The distribution shows moderate dispersion, likely due to adaptive adjustment in response to movement. Overall, these results demonstrate that symmetry, balance index, and pelvis tilt provide complementary information regarding postural alignment and stability, supporting their integration into the proposed multi-domain feature representation.

### 4.5. Upper-Body Dynamics

**Figure 12:**
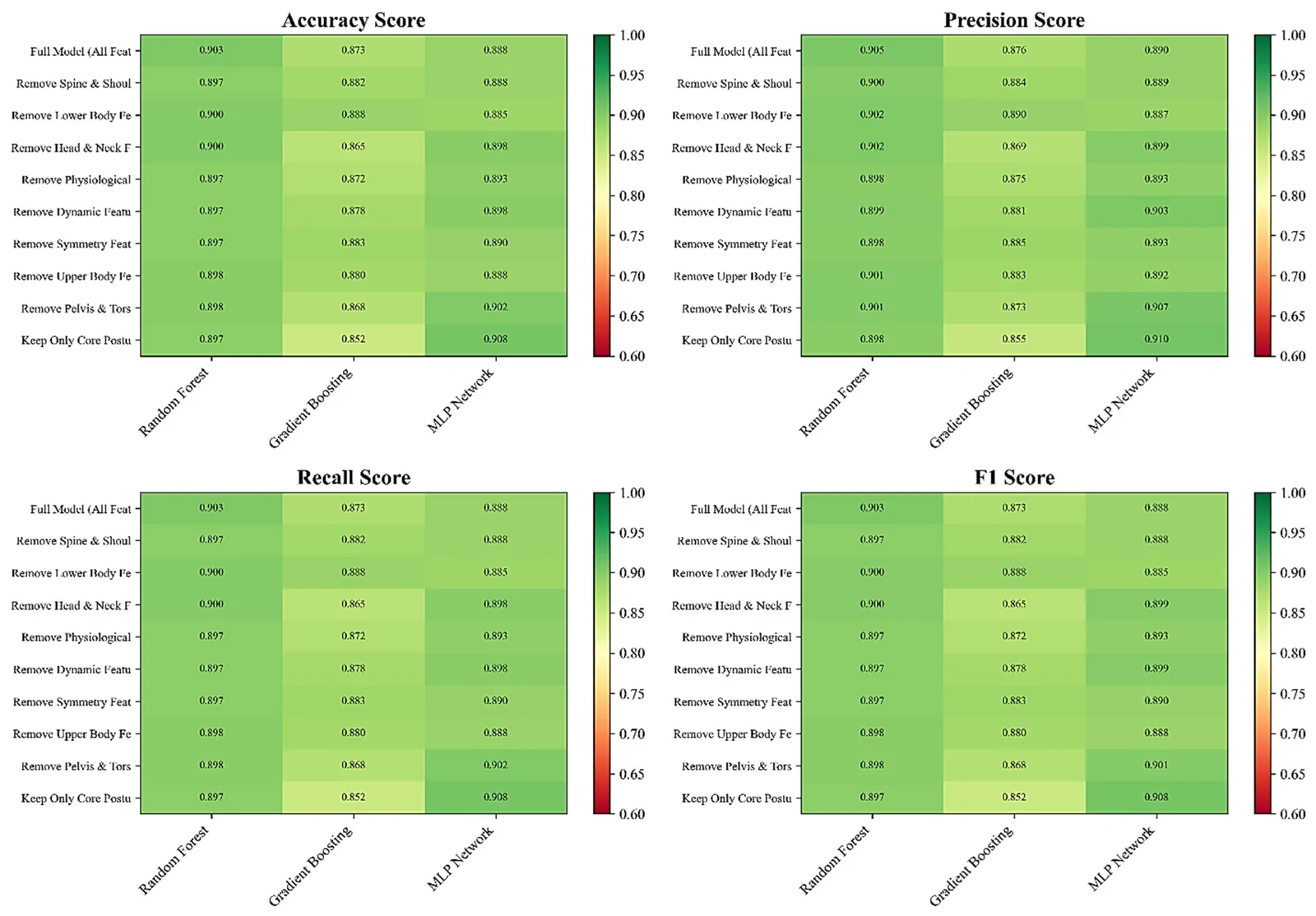
Heatmap visualization of performance comparison models.

**Figure 13:**
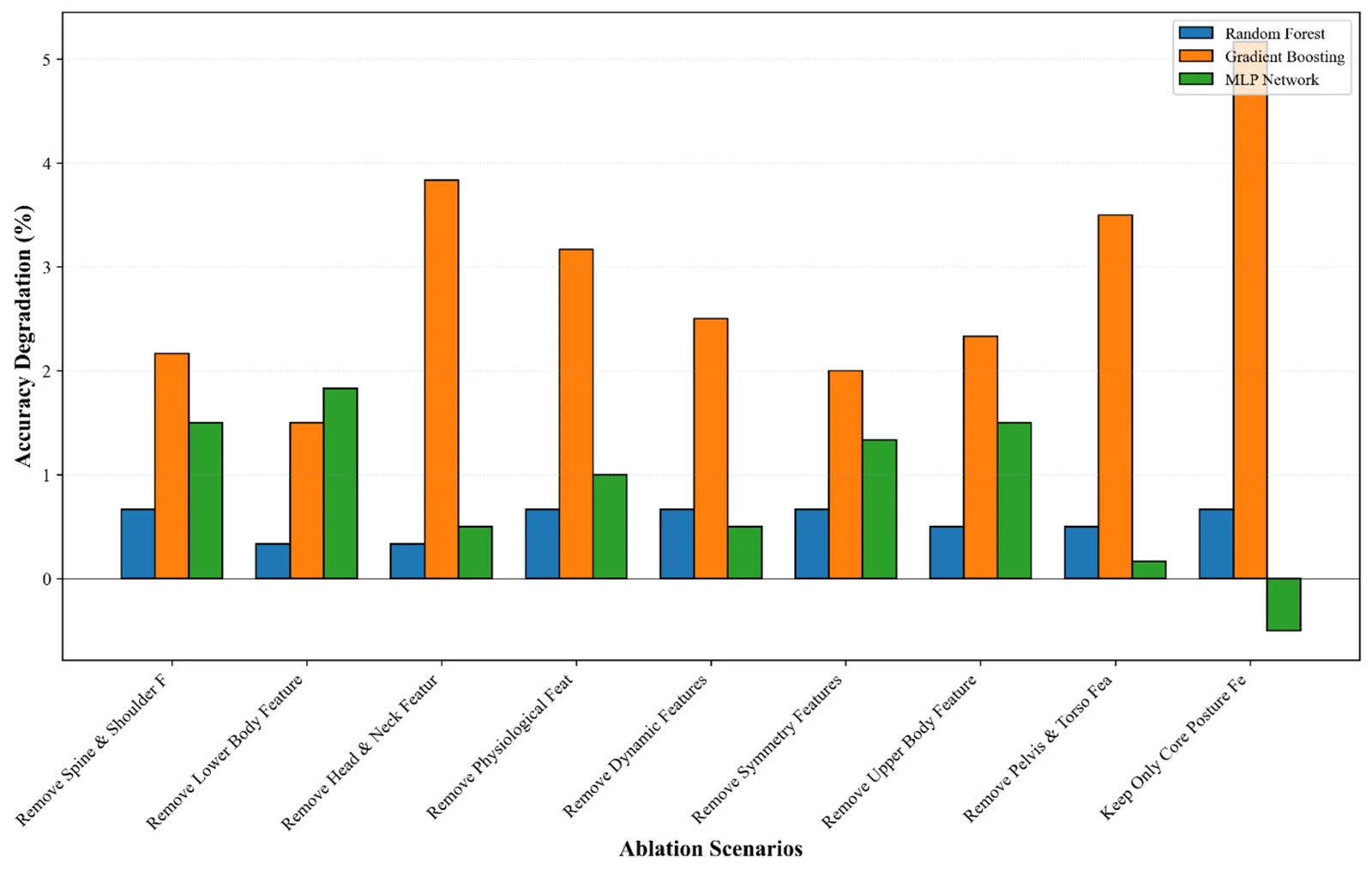
Comparative analysis of accuracy degradation under different feature ablation scenarios.

Following the postural alignment and stability analysis presented in Section 4.4, this section focuses on upper-body biomechanical dynamics. The analysis investigates upper-limb characteristics, including arm alignment, elbow angle, wrist angle, scapular retraction, and shoulder blade positioning, which provide complementary information about upper-limb coordination and structural control during exercise execution. The temporal-, statistical, and frequency-domain behaviors of these upper-body features are analyzed in Figure 9, Figure 10 and Figure 11 to characterize their contribution to posture quality assessment. The time-series analysis shows a moderate fluctuation over time, indicating a certain degree of control in the alignment of bilateral systems. It is a balanced distribution with some minor deviations, indicating that the subject is maintaining a balanced posture. Dominant low-frequency components are identified by frequency-domain analysis, which is related to natural body sway. The balance index is an indicator of the stability of the body in motion and stationary states. The overall temporal signal has smoother fluctuations than the joint-level features, suggesting overall equilibrium control. The dispersion of the distribution is moderate, and the frequency analysis shows that the low-frequency components are dominant and are mechanisms for postural stabilization. Pelvis tilt is an important structural element that affects the alignment of the spine and posture of the lower body. The time-series analysis shows moderate-amplitude periodic fluctuations, which represent the adaptive changes of posture. Controlled pelvic movement is illustrated by a relatively concentrated distribution. The frequency-domain analysis is used to determine dominant components related to repetitive motion patterns. Table 5 summarizes the statistical and dynamic properties of the features analyzed for a convenient overview. Table 6 presents the quantitative results, and Figure 12 and Figure 13 show the visual performance comparisons and degradation analysis. The results of the experiments show that the model of the Random Forest in most cases yielded stable accuracy results of around 90% in almost all ablation cases. Temporal-domain visualization illustrates movement continuity, stability, oscillatory behavior, and transient deviations that directly influence posture quality assessment.

**Figure 14:**
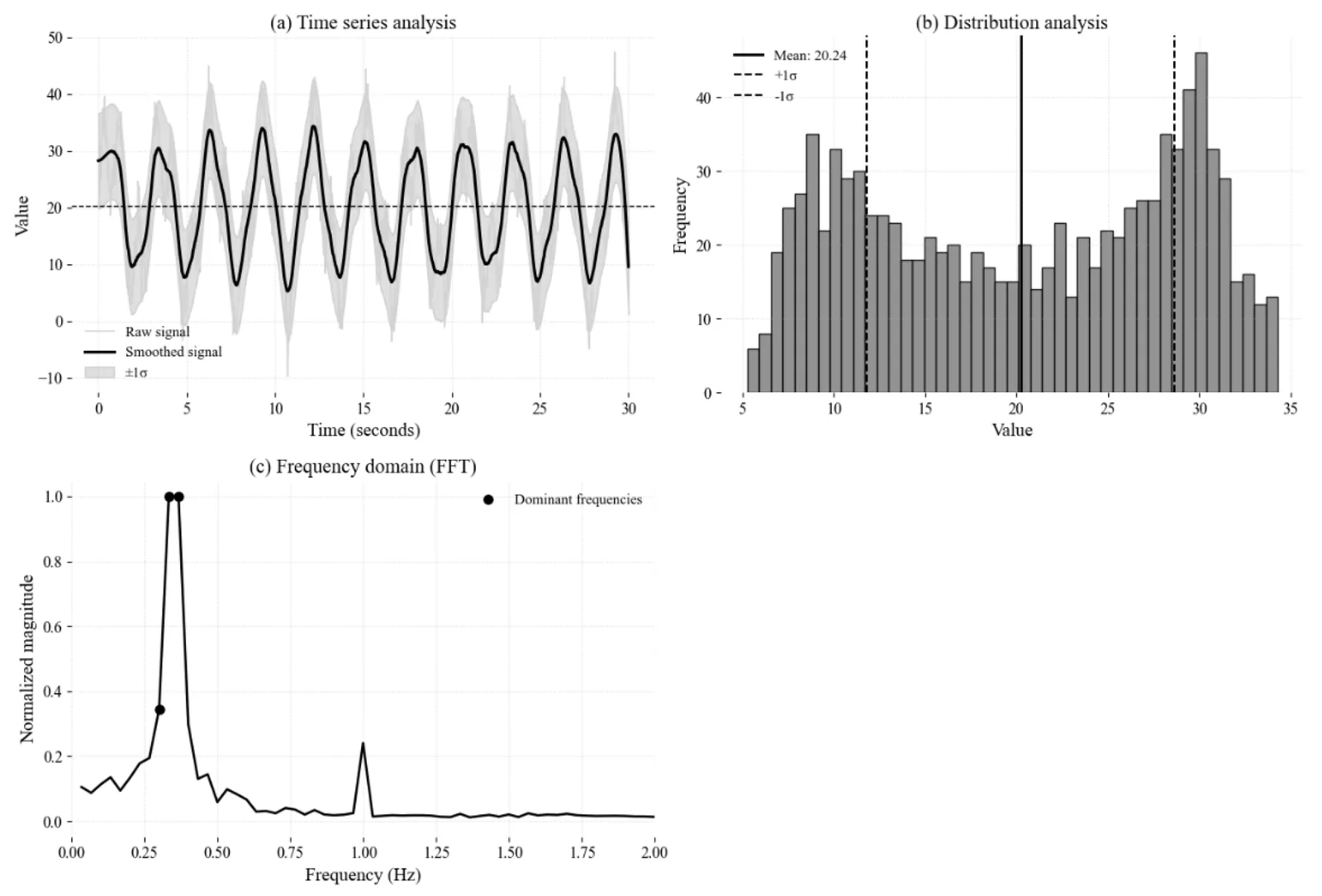
Comprehensive analysis of a representative biomechanical signal for Arm_Alignment.

**Figure 15:**
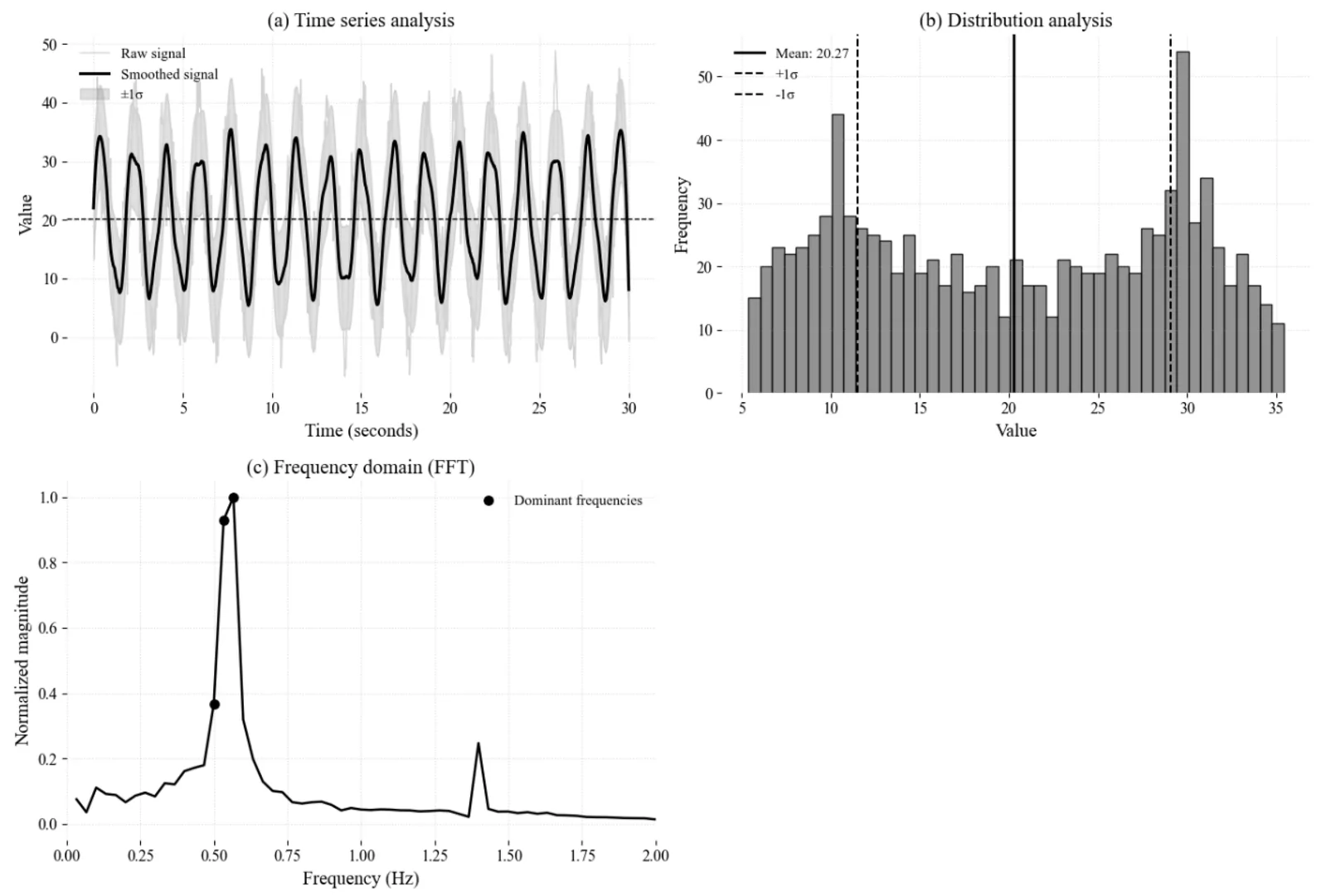
Comprehensive analysis of a representative biomechanical signal for Elbow_Angle.

**Figure 16:**
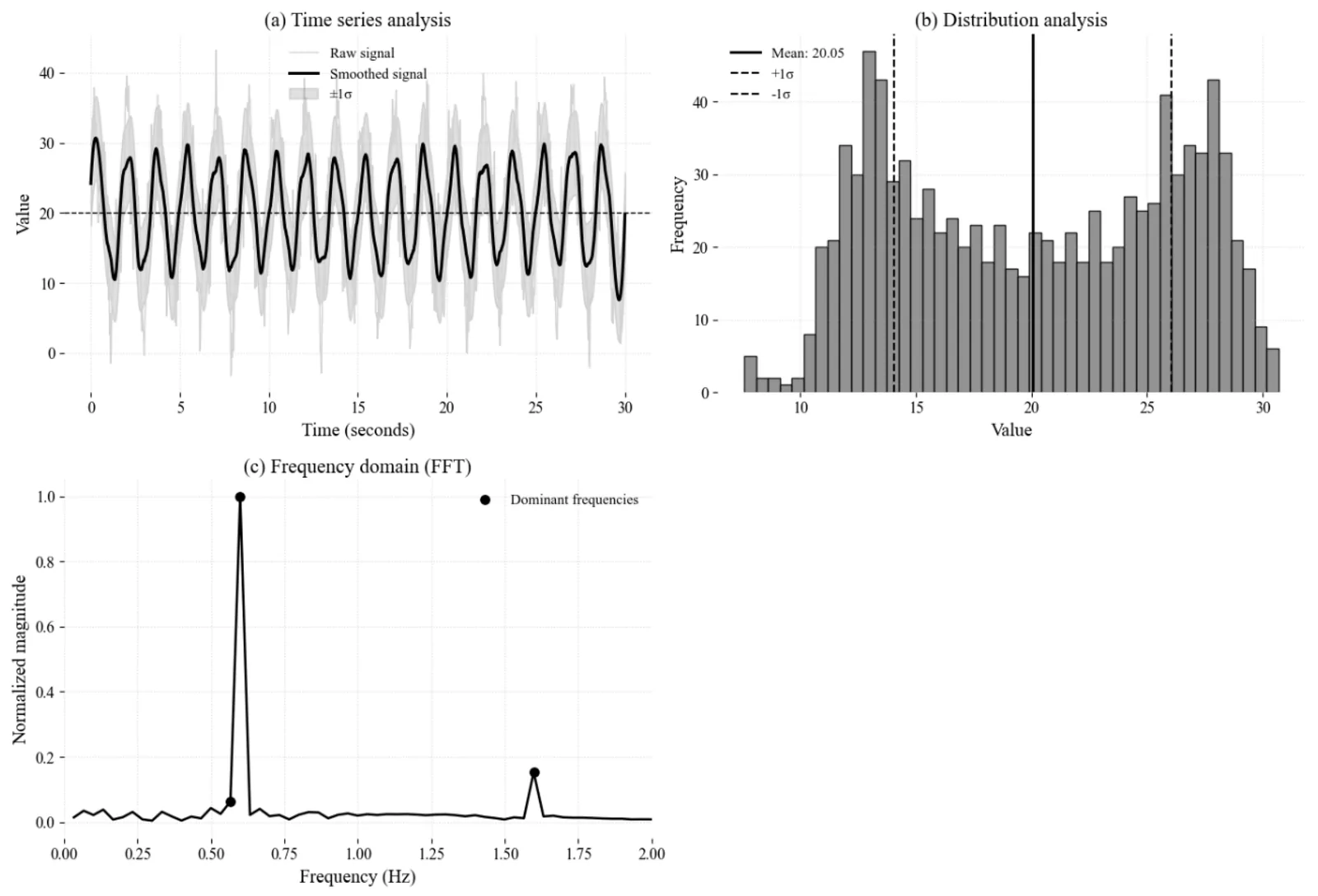
Comprehensive analysis of a representative biomechanical signal for Wrist_Angle.

**Figure 17:**
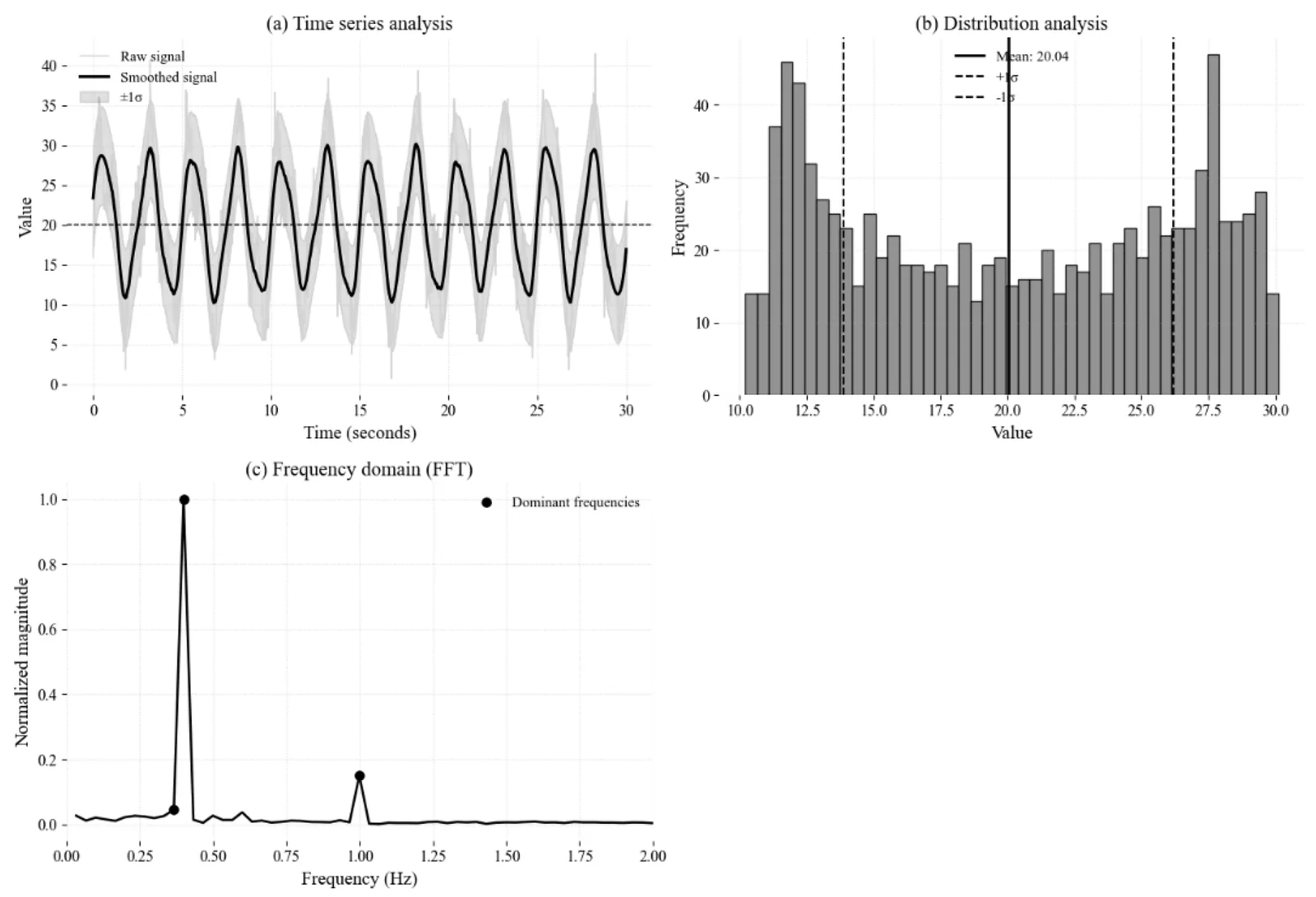
Comprehensive analysis of a representative biomechanical signal for Scapular_Retraction.

**Figure 18:**
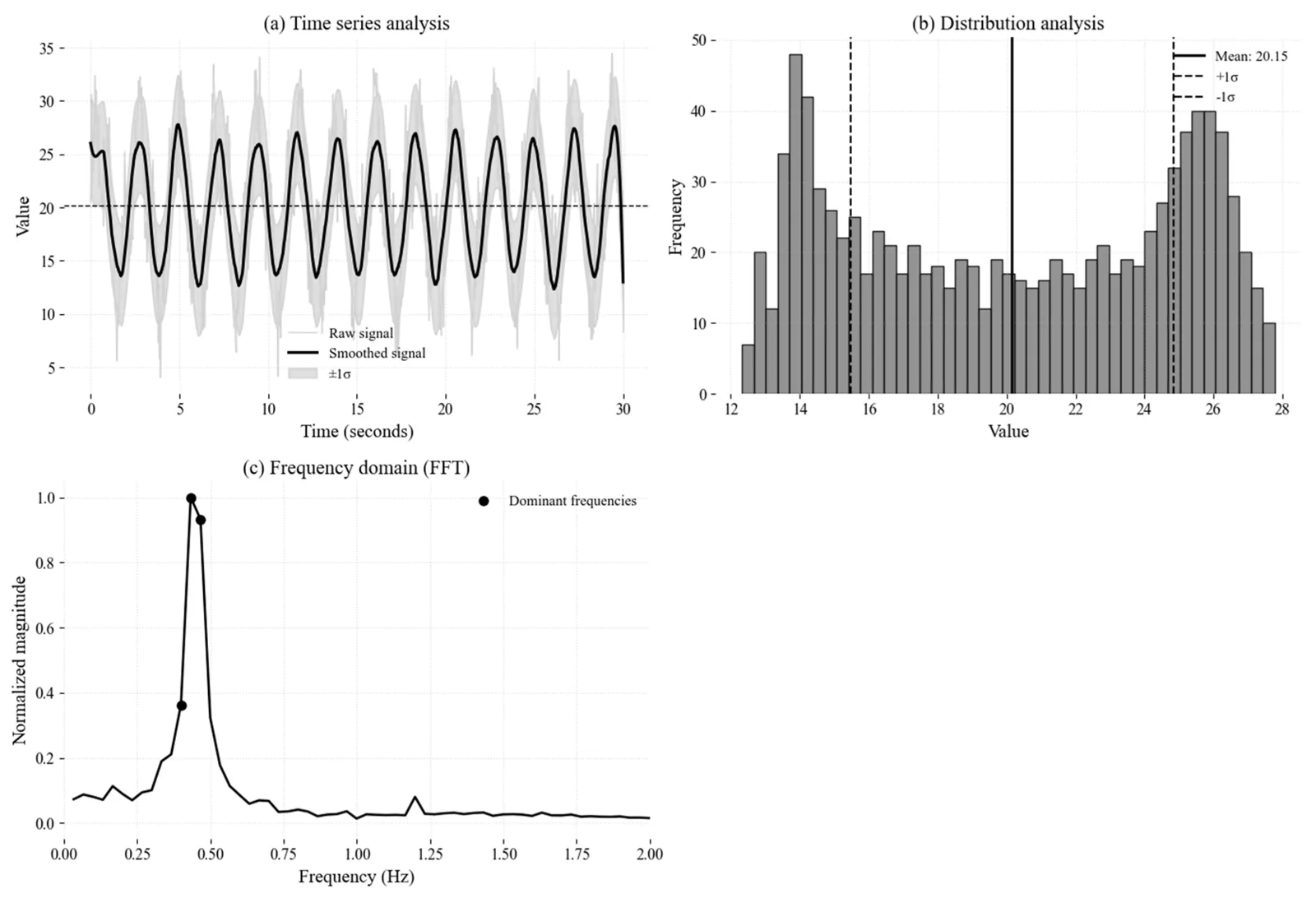
Comprehensive analysis of a representative biomechanical signal for Shoulder_Blade_Position.

The upper-limb biomechanical characteristics that are detailed in this section are arm alignment, elbow angle, wrist angle, scapular retraction and shoulder blade position. They reflect the subtle motion dynamics and structural alignment of the upper body that are key to assessing posture quality and identifying subtle deviations. The distribution is even, and the frequency analysis shows dominant low-frequency components associated with natural arm movement, as shown in Figure 14. The elbow angle is more variable as it is part of dynamic articulation. There are significant oscillations in the time series and a relatively broad distribution. The frequency-domain analysis reveals several significant peaks, indicating complex movement patterns (refer to Figure 15). The wrist angle shows fine-grained movement and high-frequency variations. The temporal signal has a high degree of fluctuation, and the distribution is wider than the proximal joints. Frequency analysis reveals both low- and mid-frequency components, including the micro-adjustments, as illustrated in Figure 16. The scapular retraction is muscle engagement in the upper back and shoulder stability. The signal is smooth and periodic but has some moderate variation. This distribution is compact, and the frequency-domain analysis shows dominant low frequencies, which are representative of stable control (Figure 17). The positioning of the shoulder blades is a sign of the alignment of the upper torso. The temporal signal exhibits controlled oscillations of low to moderate variability. The distribution is still centered, and frequency analysis reveals low-frequency motion patterns, which are stable, as seen in Figure 18.

**Table 6:** Statistical Summary of Upper-Limb Biomechanical Features.

Feature	Mean (°)	Std. Dev. (°)	Dominant Frequency (Hz)
Arm Alignment	20.4	14.3	0.3, 1.0
Elbow Angle	19.7	15.9	0.6, 1.5
Wrist Angle	20.5	14.4	0.5, 1.4
Scapular Retraction	20.5	8.2	0.6, 1.5
Shoulder Blade Position	20.1	6.7	0.6, 1.4

### 4.6. Lower-Body Dynamics

**Figure 19:**
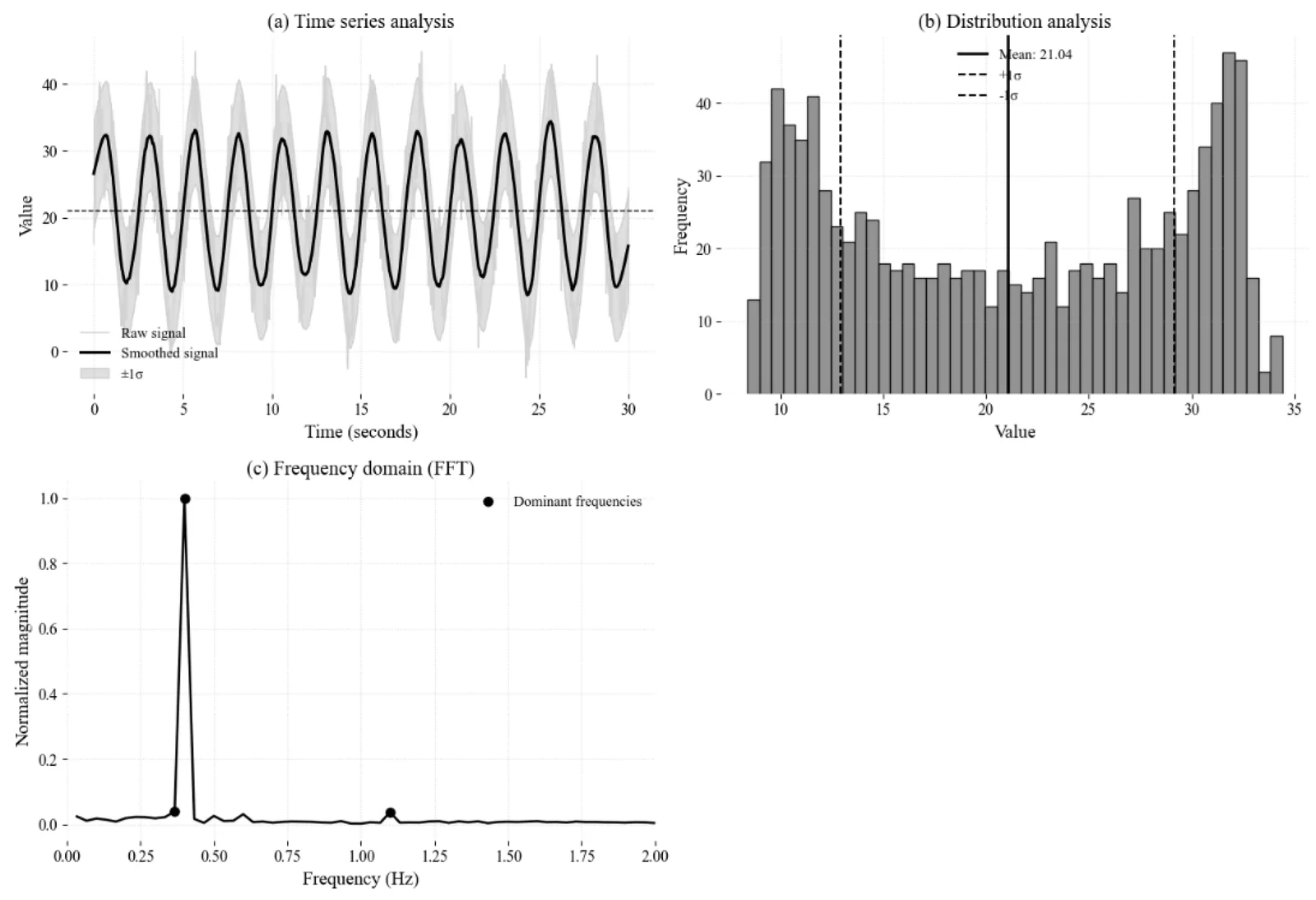
Comprehensive analysis of a representative biomechanical signal for Leg_Alignment.

**Figure 20:**
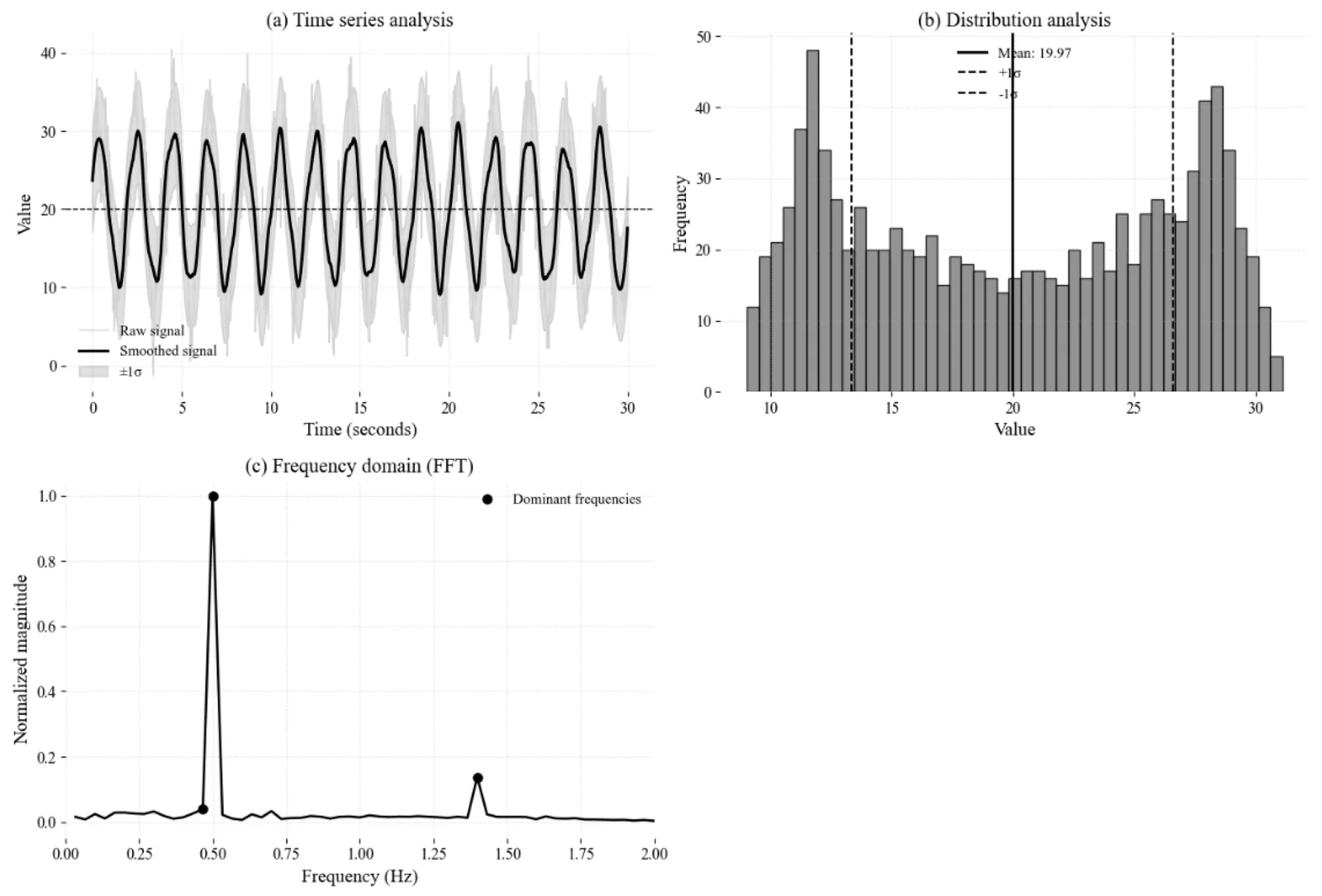
Comprehensive analysis of a representative biomechanical signal for Ankle_Angle.

This section emphasizes the basic biomechanical properties of the lower leg (leg alignment and ankle angles), which are important for balance, locomotion and postural stability. These features are used to represent the structural alignment and any fast corrective movements needed during posture adjustment. Leg alignment is the positioning and coordination of the lower limbs. The time-series analysis shows that there are periodic oscillations of relatively high amplitude, which means that they are actively involved in maintaining balance. There is moderate to high variability in the distribution, and frequency analysis indicates dominant components corresponding to gait like patterns and repetitive motion cycles, as exhibited in Figure 19. The ankle angle shows more frequent oscillations than other lower-body characteristics, and this angle is important for fine-grained balance corrections (temporal signal: high fluctuations; distribution: high spread). Dominant higher-frequency components in the frequency-domain analysis are related to the micro-adjustments of posture stabilization, as displayed in Figure 20.

### 4.7. Physiological Signal Analysis

**Figure 21:**
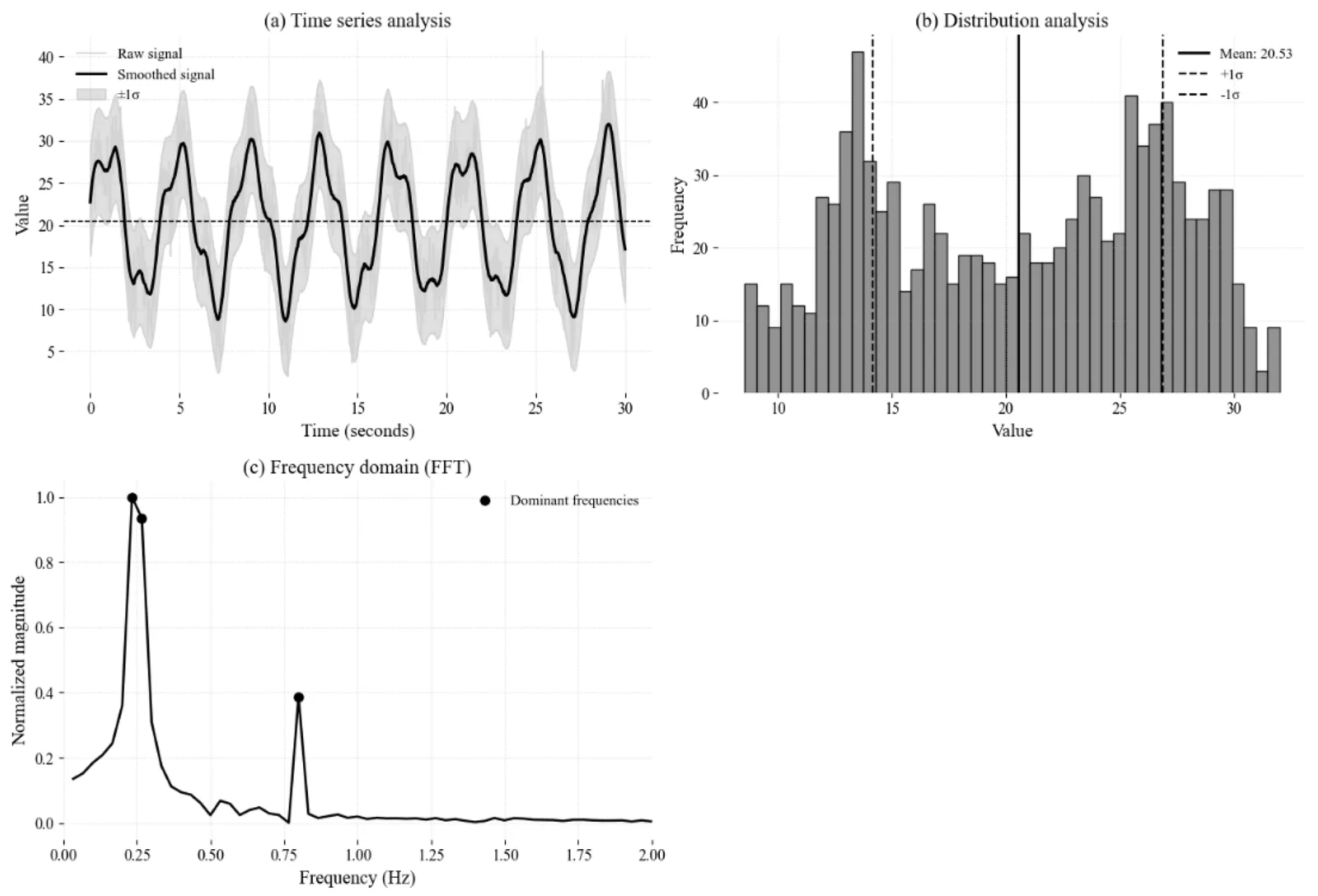
Comprehensive analysis of a representative biomechanical signal for Breathing_Rate.

**Figure 22:**
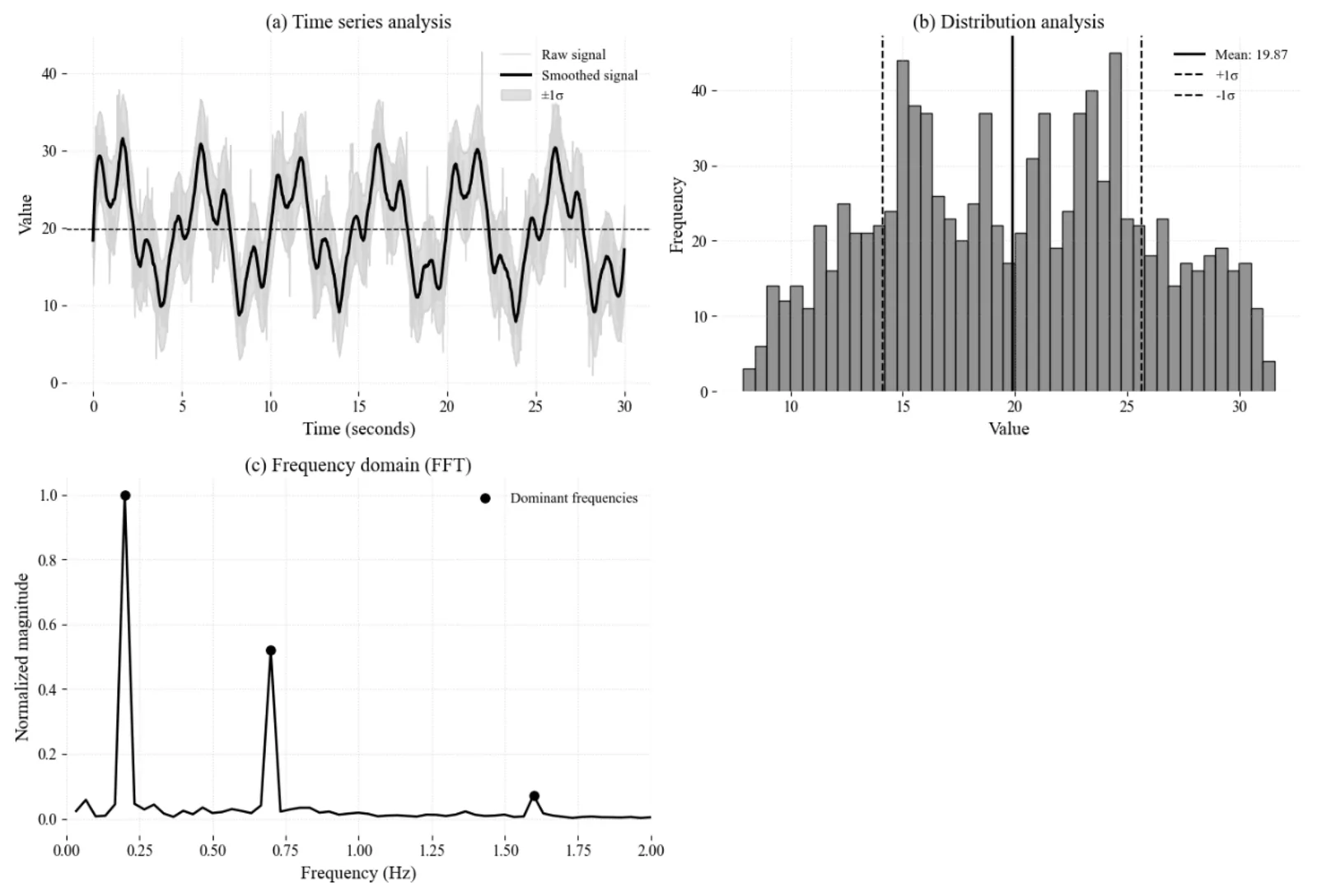
Comprehensive analysis of a representative biomechanical signal for Heart_Rate_Variability.

**Figure 23:**
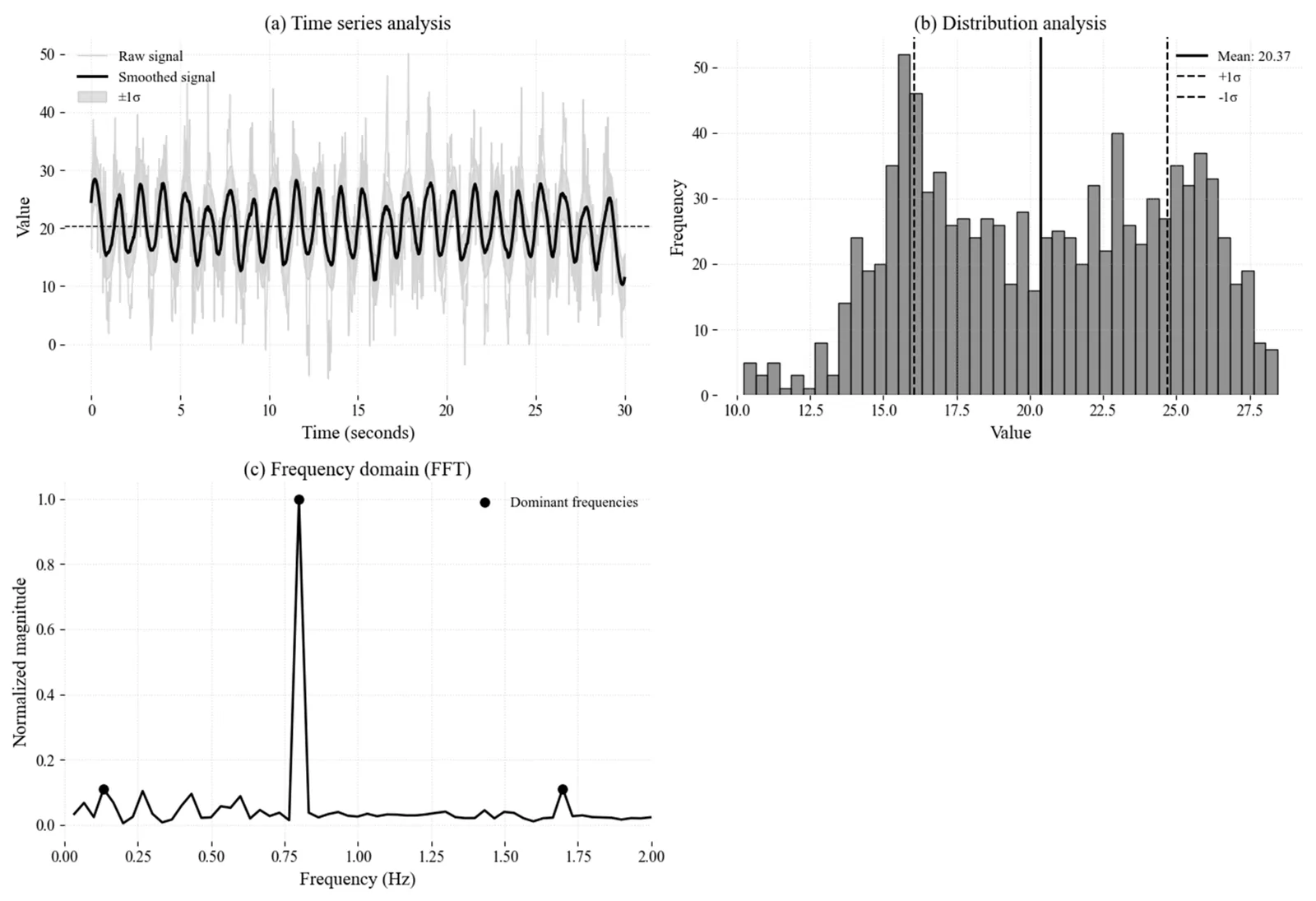
Comprehensive analysis of a representative biomechanical signal for Muscle_Activation.

**Figure 24:**
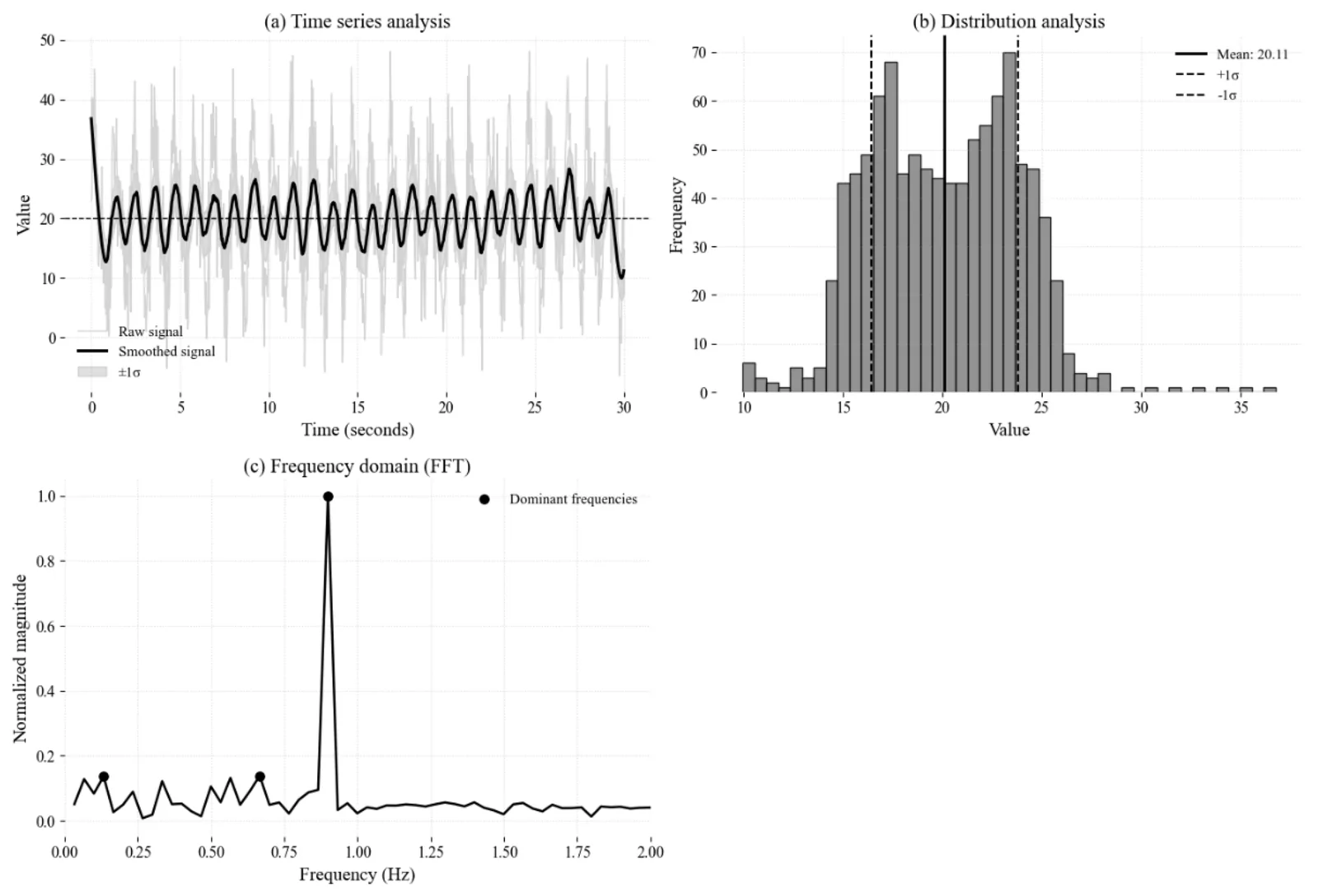
Comprehensive analysis of a representative biomechanical signal for Joint_Velocity.

**Figure 25:**
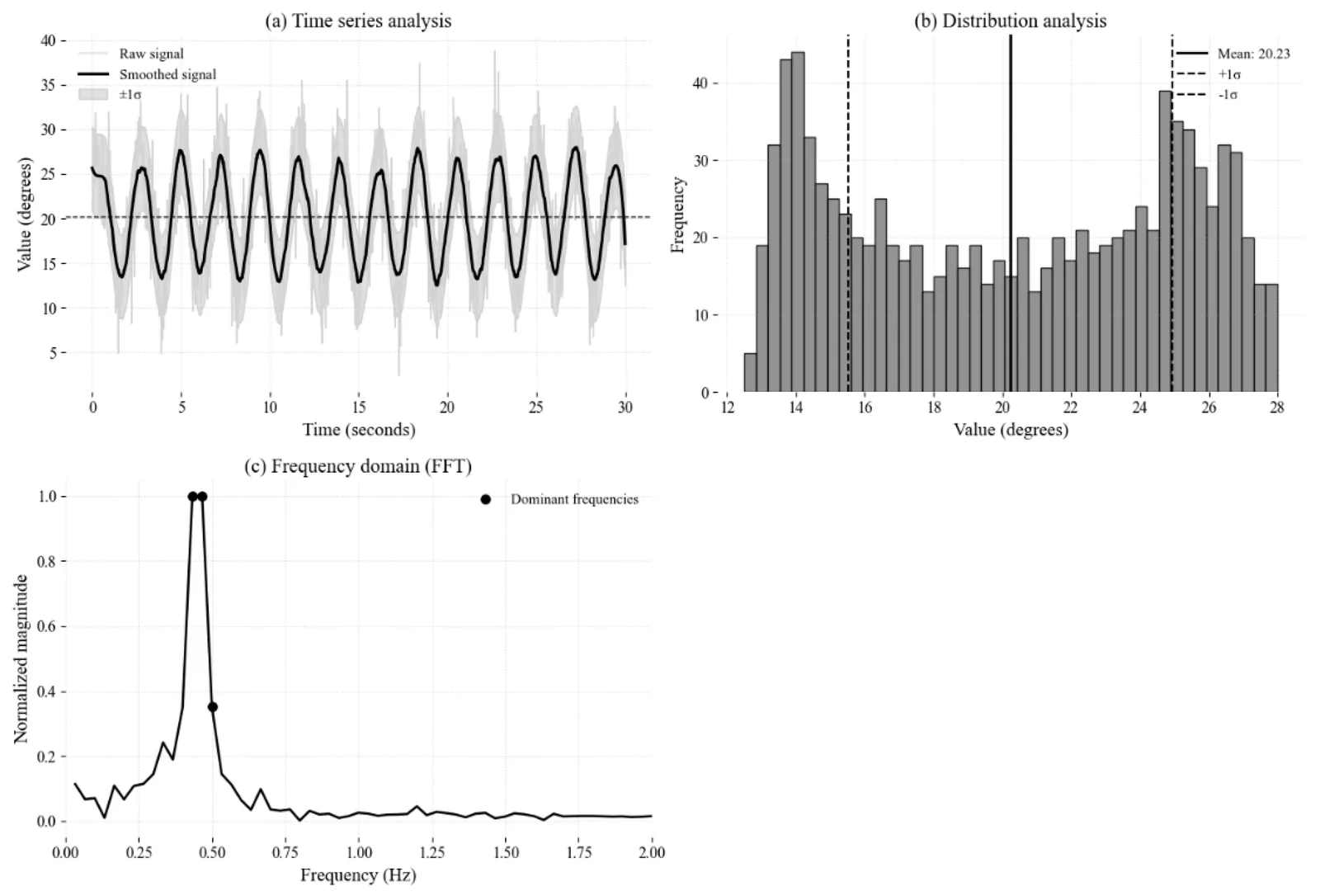
Comprehensive analysis of a representative biomechanical signal for Thoracic_Curve.

**Figure 26:**
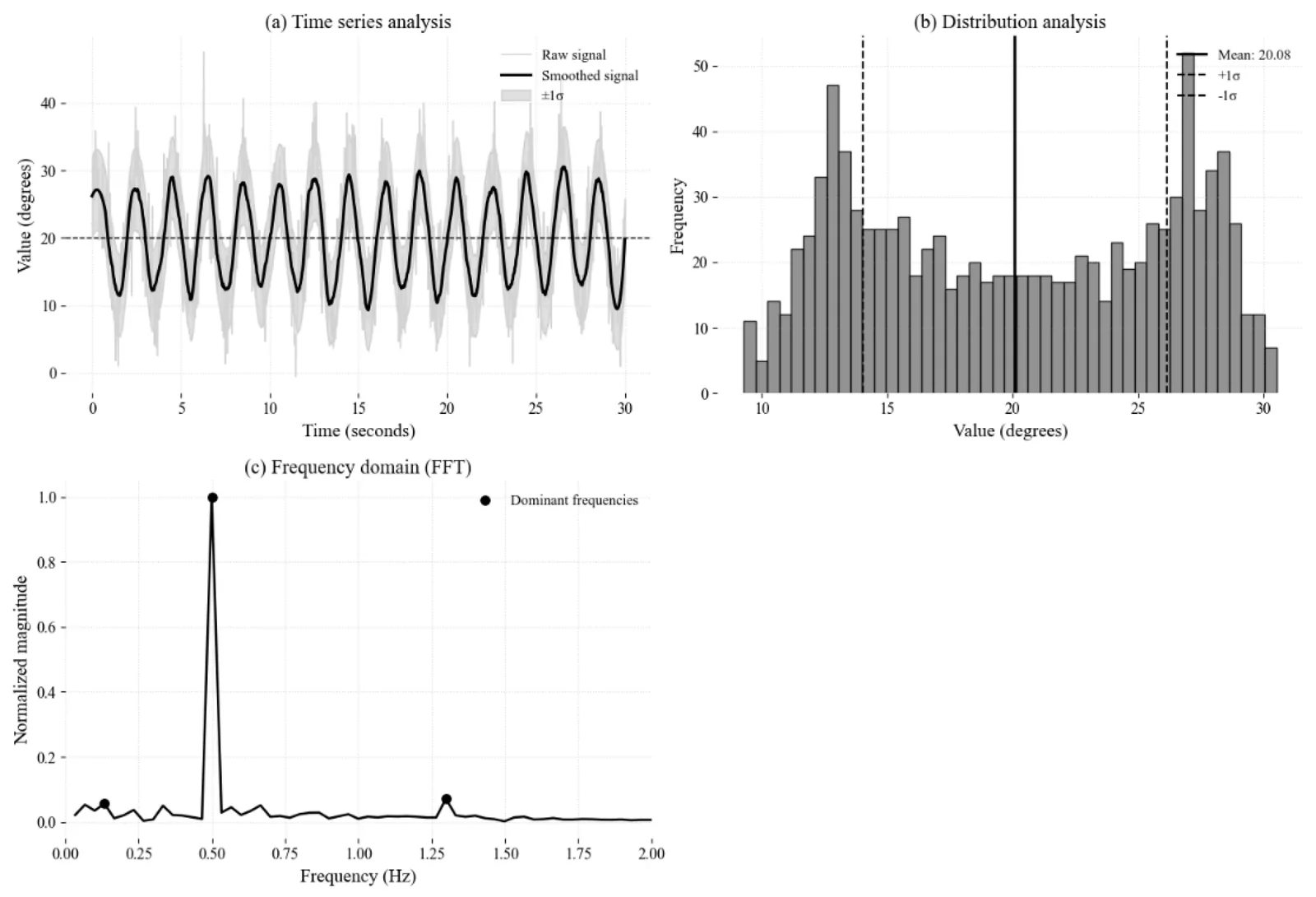
Comprehensive analysis of a representative biomechanical signal for Lumbar_Curve.

In this section, physiological and neuromuscular signals, such as breathing rate, heart rate variability (HRV), and muscle activation, which complement the information of internal physiological state and control mechanism during posture maintenance, are examined. The breathing rate signal shows smooth, low-frequency oscillations which appear to represent respiratory cycles. The distribution is tight, suggesting the physiological behavior is stable [30,31]. The dominant components in the expected respiratory range are confirmed by frequency analysis, as seen in Figure 21. HRV shows complex multiple frequency dynamics that correspond to the activity of the autonomic nervous system. The temporal signal exhibits irregular fluctuations, and the distribution is broader than breathing rate. Multiple dominant peaks are seen in frequency-domain analysis, representing physiological regulatory processes, as illustrated in Figure 22. Neuromuscular activity can be seen in muscle activation signals as high-frequency oscillations in bursts: high variability (broad distribution) and rapid variations (temporal signal). The peaks that are prominent in the frequency analysis are associated with contraction cycles, as illustrated in Figure 23. Advanced motion and structural characteristics such as joint velocity, thoracic curvature and lumbar curvature are emphasized in this section, offering additional information regarding dynamic movement patterns and spinal alignment. These features allow us to analyze both movement transitions and structural posture stability, providing a multi-scale representation of posture behavior. Joint velocity is the measurement of how quickly the position of a joint changes, which indicates the dynamics of the motion. The temporal signal is very dynamic and abrupt, indicating that movement adjustments are very fast. The distribution is wide and the frequency analysis shows dominant high-frequency components related to dynamic motion patterns, as in Figure 24. The thoracic curve is the alignment of the upper spine and shows moderate variability in its oscillatory behavior. The temporal signal is stable, and the distribution is not too wide. Frequency-domain analysis shows dominant low-frequency components, which are associated with controlled posture maintenance, as in Figure 25. The lumbar curve is more variable than the thoracic curve, because it is used for dynamic postural adjustments. The temporal signal has moderate oscillations and the distribution is wider. Dominant components identified using frequency analysis are related to adaptive movement patterns, as illustrated in Figure 26. A complete Statistical Summary of Biomechanical, Physiological and Structural Features is provided in Table 7.

### 4.8. Advanced Motion and Structural Analysis

In this section, some advanced biomechanical parameters are presented and analyzed, which reflect not only the dynamic motion characteristics but also the structural motion characteristics of the spine, including the joint velocity, thoracic curvature and lumbar curvature. These features provide a more abstract representation of posture, incorporating both kinematic change and anatomical alignment, and can provide a more comprehensive representation of human movement and stability. A comparative analysis of the features studied emphasizes the differences and complementary role of the features in the assessment of posture [32]. The velocity of the joints has the greatest range and high-frequency content, making it ideal to capture the high-velocity dynamics of movement and transient transitions in instability. The thoracic curvature, however, shows the most consistent behavior and suggests structural stability and consistent upper-body alignment. The combination of these characteristics is important: for sudden changes in posture and instability, dynamic characteristics are important; for spinal alignment and quality of posture, structural characteristics are important. The use of both motion and structural data improves the robustness and the ability of the model to predict the posture assessment [33].

The hybrid learning architecture also improves the system by incorporating ensemble-based techniques and neural networks, allowing for the advantages of each approach to be utilized. Ensemble models were found to be useful to obtain stability and robustness, particularly if the feature distributions are heterogeneous, while neural models are useful for learning complex nonlinear relationships. The synergy leads to better transfer of knowledge to other posture patterns and user situations. These are good things, but there are a few drawbacks. The current evaluation used a particular set of data and conditions; this may not adequately represent the variation found in different populations in the real world. In addition, the model is performing well but can be further enhanced by using larger datasets, custom calibration features and real-time deployment factors.

To assess the importance of each feature group related to posture for classification, comprehensive ablation experiments were carried out for three machine learning models, namely Random Forest (RF), Gradient Boosting (GB), and MLP Network. The experiments systematically excluded certain categories of features, such as spine and shoulder features, lower-body features, head and neck features, physiological features, dynamic features, symmetry features, upper-body features, and pelvis and torso features. Furthermore, a compact version of the feature configuration with only the basic posture features was explored. 

Frequency-domain analysis using FFT identifies dominant movement frequencies, tremors, compensatory movements, and micro-adjustments that are not observable in raw temporal signals alone.

Distributional analysis provides insights into signal variability, consistency, and deviation patterns, which are closely related to movement quality and postural control.

Peak-based analysis enables the identification of sudden posture transitions, instability events, and abnormal movement corrections that contribute significantly to classification decisions.

Feature interpretability and explainability are enhanced by providing visual evidence of how individual biomechanical signals contribute to the final prediction, an important consideration for clinical rehabilitation and human-centered AI systems.

Clinical and rehabilitation relevance requires transparent and interpretable models rather than black-box predictions. The presented visualizations facilitate understanding by clinicians, therapists, and rehabilitation specialists regarding the physiological basis of the model decisions.

Validation of feature engineering choices is supported through these analyses by demonstrating that the extracted time-domain, frequency-domain, and peak-related descriptors capture meaningful biomechanical information rather than arbitrary statistical patterns. Table 8 presents a comprehensive comparative evaluation of the proposed EETANet framework against a diverse set of posture assessment approaches, including traditional machine learning methods, deep learning architectures, wearable sensor-based systems, pose estimation techniques, and rehabilitation assessment models. The comparison was conducted using standard classification metrics, namely accuracy, precision, recall, F1-score, area under the ROC curve (AUC), and the number of input features. Among all evaluated approaches, the proposed Random Forest-based EETANet configuration achieved the highest overall performance, attaining an accuracy of 90.33%, a precision of 90.49%, an F1-score of 90.33%, and an AUC of 98.93%. These results demonstrate the strong discriminative capability and robustness of the proposed framework for posture quality classification. Similarly, the MLP-based implementation of the proposed framework delivered competitive results, achieving an accuracy of 88.83% and an AUC of 98.89%, highlighting the effectiveness of nonlinear feature learning in capturing complex biomechanical relationships. Among the conventional machine learning methods, both Support Vector Machines (SVMs) and K-Nearest Neighbors (KNNs) achieved competitive performance, with accuracies of 87.17% and 87.67%, respectively. In contrast, Logistic Regression exhibited substantially lower predictive performance, achieving only 60.83% accuracy, indicating that linear decision boundaries are insufficient to model the nonlinear and heterogeneous nature of posture dynamics. Regarding deep learning approaches, the CNN model achieved an accuracy of 87.33%, demonstrating performance comparable to Gradient Boosting and other high-performing traditional methods. However, the standalone LSTM and Transformer architecture yielded considerably lower accuracies of 33.73% and 35.25%, respectively. This observation suggests that sequence-based architecture may not fully exploit the discriminative information available in the engineered feature representations used in the current study and may require larger datasets or raw temporal sequences for effective learning that are illustrated in Table 9. Furthermore, wearable sensor-based approaches achieved strong performance with an accuracy of 89.17% while utilizing only 10 features, demonstrating the effectiveness of compact sensor configurations for practical deployment. Conversely, pose estimation approaches based on OpenPose achieved significantly lower accuracy (29.00%), emphasizing the limitations of relying exclusively on visual skeletal information for comprehensive posture evaluation. These findings collectively highlight the importance of integrating multimodal biomechanical and physiological features to achieve reliable and robust posture assessment in rehabilitation and fitness monitoring applications.

The MLP model achieved competitive or better performance on most of the evaluation metrics in multiple scenarios, especially in the scenario with reduced feature dimensionality, namely when accuracy, precision, recall, and F1-score were used. This behavior illustrates the effectiveness of neural networks to learn compact and discriminative feature representations. The last important thing that can be noted from the ablation analysis is that the models of Random Forest and MLP did not experience significant performance drops when ablating lower-body features and head and neck features. This result indicates that the classification of posture could be based more on the global body alignment and core posture dynamics rather than on the individual body parts measurements. On the other hand, the degradation of the Gradient Boosting model was relatively large when the pelvis and torso features were removed, suggesting that the central body posture information is crucial to the Gradient Boosting model.

## 5. Conclusions

We proposed a novel human-centric ambient intelligent system for automated posture assessment, named EETANet (Edge-Enhanced Temporal Attention Network), in the rehabilitation and fitness monitoring area. The proposed system aims to address this need by providing an end-to-end system that transforms multivariate motion signals into interpretable and meaningful posture quality predictions in autonomous and home-based environments, thereby enabling accurate and reliable assessment of posture. It was developed using a multi-domain feature extraction approach, that is, features extracted in the time domain and frequency domain (FFT and peak detection), which are effective in capturing steady-state posture characteristics and transient instability patterns. Moreover, the use of temporal attention mechanisms further improves the model’s capability of capturing motion continuity and detecting subtle posture deviations, which are crucial for real-world applications. The hybrid learning structure of ensemble-based models and neural models is more robust and generalizes and adapts in heterogeneous feature spaces.

The proposed framework has some potential but can be expanded in a couple of ways to be more capable:

(i) The model can be applied to edge devices, such as wearable sensors and mobile platforms, to enable low-latency, real-time posture monitoring.
(ii) Incorporating adaptive learning processes to adapt the system for a particular user, considering differences in body structure, fitness and rehabilitation needs.
(iii) Scaling up the framework to incorporate other data sources, such as vision-based inputs, depth sensors, or electromyography (EMG) signals, for improved accuracy and robustness. While it has its advantages, there are some drawbacks and issues to consider:
i. The model may yield varying results if applied to a different set of users, as there may be variations in the quality of the sensors, the environment, and the diversity of the users.
ii. This current evaluation may not fully capture the true variability in the field, and more and varied datasets are needed.
iii. Multi-domain features and hybrid learning models could result in higher computational needs, impacting deployment on resource-constrained devices.
iv. Physiological and motion data taken in uncontrolled environments can be noisy and incomplete, which can impact system reliability.
v. Guaranteeing user engagement and proper sensor positioning is still practical in home-based systems.

Experimental results demonstrate that the proposed EETANet framework achieves promising performance for multi-level posture quality classification using a compact set of core biomechanical features. However, the current evaluation was conducted using synthetically generated motion signals, and, therefore, the findings should be interpreted within the scope of the experimental setting. Although the results demonstrate the feasibility and effectiveness of the proposed approach, additional validation is required before drawing conclusions regarding its deployment in real-world rehabilitation environments. Future work will focus on validating EETANet using data collected from real participants, wearable sensing devices, and clinical rehabilitation datasets to evaluate its robustness and generalizability under practical conditions. We also plan to investigate cross-subject and cross-device generalization, incorporate multimodal sensing modalities, and assess the framework in real-time rehabilitation and home-based healthcare applications. These future developments will further strengthen the clinical applicability, reliability, and scalability of the proposed framework for intelligent posture assessment and personalized rehabilitation support. Future investigations will include a systematic component-level ablation study to independently quantify the contributions of the major EETANet components. The complete framework will be compared with controlled variants, excluding Savitzky–Golay smoothing, frequency-domain features, peak-based features, edge enhancement, and temporal attention. Maintaining identical data partitions, training configurations, and evaluation protocols across these variants will enable a more rigorous assessment of the contribution of each component. Such experiments will help distinguish the effects of preprocessing, multi-domain representation, and architectural mechanisms and provide stronger empirical evidence regarding their respective roles in posture quality classification.

## Figures and Tables

**Table 1:** Description of the 24 biomechanical and physiological features.

Category	Feature Name	Description
**Joint Kinematics**	Knee, Hip, Ankle Angles	Measures flexion/extension cycles during lower-body exercises.
	Elbow, Wrist, Shoulder Angles	Monitors limb extension and distal upper limb movement fluency.
	Head Position and Neck Angle	Tracks alignment of the cervical spine and rhythmic head adjustments.
	Joint Velocity	Analyzes speed of movement to detect jerky or unstable motion patterns.
**Spinal Alignment**	Thoracic and Lumbar Curves	Identifies postural collapse or excessive strain in the upper/lower back.
	Spine Angle and Pelvis Tilt	Global metrics for torso verticality and core orientation.
	Scapular Retraction	Measures engagement of the back muscles during bracing phases.
**Dynamic Stability**	Posture Symmetry	Completes the overall posture and differentiates between “Good” and “Fair” posture.
	Balance Index	Multi-sensor fusion metric for separating out adjustments from noise.
	Stability Score	Synthesizes overall poise to distinguish between “Good” and “Fair” posture.
**Physiological**	Muscle Activation	Intensity of physiological engagement and primary contraction cycles.
	Core Engagement	Tracks abdominal/back bracing required for postural foundations.
	Breathing and Heart Rate	Monitors cardiovascular response and rhythm under exercise stress.
	Heart Rate Variability (HRV)	Detects fatigue-related postural decline and physiological context.
**Raw Sensor Data**	Accel (X, Y, Z)	Raw tri-axial acceleration capturing linear motion dynamics.
	Gyro (X, Y, Z)	Raw tri-axial angular velocity capturing rotational movement.

**Table 2:** Mapping of Posture Quality Score (PQS) intervals to the corresponding five posture quality labels.

PQS Interval	Posture Quality Label
**0.00–0.20**	Very Poor
**0.20–0.40**	Poor
**0.40–0.60**	Fair
**0.60–0.80**	Good
**0.80–1.00**	Excellent

**Table 3:** Distribution of the five posture quality classes across 5000 synthetic samples.

Posture Quality Class	Samples	Percentage
**Very Poor**	55	1.10%
**Poor**	1021	20.42%
**Fair**	2617	52.34%
**Good**	1250	25.00%
**Excellent**	57	1.14%
**Total**	5000	100.00%

**Table 4:** Statistical Summary of Core Biomechanical Features.

Feature	Mean (°)	Std. Dev. (°)	Dominant Frequency (Hz)
Spine Angle	20.5	6.5	0.5
Neck Angle	20.0	11.0	0.5, 1.3
Hip Angle	20.3	8.5	0.6
Knee Angle	20.7	11.3	0.4, 1.0

**Table 5:** Statistical Summary of Postural Alignment and Stability Features.

Feature	Mean (°)	Std. Dev. (°)	Dominant Frequency (Hz)
Posture Symmetry	20.6	6.6	0.8
Balance Index	20.4	7.0	0.7
Pelvis Tilt	20.5	8.0	0.5

**Table 7:** Comprehensive Statistical Summary of Biomechanical, Physiological, and Structural Features.

Category	Feature	Mean	Std. Dev.	Dominant (Hz)
**Lower Body Features**	Leg Alignment	20.8	14.8	0.4, 1.1
	Ankle Angle	20.6	15.5	0.5, 1.4
**Physiological Signals**	Breathing Rate	20	6.5	0.2–0.8
	Heart Rate Variability	20	10.5	0.2–1.6
	Muscle Activation	20	15.8	0.8–2.0
**Advanced Motion Features**	Joint Velocity	20.9	18.5	0.9–2.0
**Spinal Structural Features**	Thoracic Curve	20.5	6.8	0.4–1.2
	Lumbar Curve	20.6	9.5	0.5–1.3

**Table 8:** Performance under different feature ablation scenarios.

Feature Configuration	Model	Accuracy (%)	Precision (%)	Recall (%)	F1-Score (%)	No. Features
**Full Model (All Features)**	RF	90.33	90.49	90.33	90.33	24
	GB	87.33	87.63	87.33	87.35	24
	MLP	88.83	88.96	88.83	88.81	24
**Remove Spine & Shoulder**	RF	89.67	89.97	89.67	89.66	20
	GB	88.17	88.42	88.17	88.18	20
	MLP	88.83	89.92	88.83	88.79	20
**Remove Lower Body**	RF	90.00	90.21	90.00	90.00	24
	GB	88.83	88.99	88.83	88.82	24
	MLP	88.50	88.70	88.50	88.48	24
**Remove Head & Neck**	RF	90.00	90.23	90.00	90.00	24
	GB	86.50	86.86	86.50	86.49	24
	MLP	89.83	89.95	89.83	89.85	24
**Remove Physiological Features**	RF	89.67	89.81	89.67	89.67	24
	GB	87.17	87.49	87.17	87.18	24
	MLP	89.33	89.33	89.33	89.28	24
**Remove Dynamic Features**	RF	89.67	89.95	89.67	89.67	24
	GB	87.83	88.15	87.83	87.84	24
	MLP	89.83	90.32	89.83	89.85	24
**Remove Symmetry Features**	RF	89.67	89.77	89.67	89.66	24
	GB	88.33	88.47	88.33	88.34	24
	MLP	89.00	89.34	89.00	88.97	24
**Remove Upper Body**	RF	89.83	90.11	89.83	89.83	24
	GB	88.00	88.26	88.00	87.97	24
	MLP	88.83	89.17	88.83	88.84	24
**Remove Pelvis & Torso**	RF	89.83	90.05	89.83	89.83	24
	GB	86.83	87.29	86.83	86.83	24
	MLP	90.17	90.67	90.17	90.15	24
**Keep Only Core Posture Features**	RF	89.67	89.82	89.67	89.67	5
	GB	85.17	85.47	85.17	85.17	5
	**MLP**	**90.83**	**90.99**	**90.83**	**90.84**	**5**

**Table 9:** Comparative performance analysis of the proposed EETANet framework against traditional machine learning, deep learning, wearable sensing, pose estimation, and rehabilitation assessment approaches using accuracy, precision, recall, F1-score, AUC, and feature dimensionality metrics.

Model	Category	Accuracy (%)	Precision (%)	Recall (%)	F1-Score (%)	AUC	No. of Features
**Random Forest (Proposed)**	Proposed Framework	**90.33**	**90.49**	**90.33**	**90.33**	**98.93**	24
**Gradient Boosting (Proposed)**	Proposed Framework	87.33	87.63	87.33	87.35	97.95	24
**MLP Network (Proposed)**	Proposed Framework	88.83	88.96	88.83	88.81	98.89	24
**SVM**	Traditional ML	87.17	87.43	87.17	87.18	98.44	24
**Logistic Regression**	Traditional ML	60.83	60.87	60.83	60.67	87.43	24
**K-Nearest Neighbors**	Traditional ML	87.67	87.95	87.67	87.64	98.38	24
**Decision Tree**	Traditional ML	85.17	85.64	85.17	85.17	90.41	24
**XGBoost (Fallback)**	Traditional ML	87.33	87.63	87.33	87.35	97.95	24
**Wearable Sensor (IMU)**	Wearable Sensors	89.17	89.38	89.17	89.14	—	10
**Pose Estimation (OpenPose)**	Pose Estimation	29.00	25.88	29.00	26.08	—	33
**Rehabilitation Assessment**	Rehabilitation Assessment	78.50	78.65	78.50	78.51	—	15
**LSTM Network**	Deep Learning	33.73	19.04	33.73	18.95	—	24
**CNN Network**	Deep Learning	87.33	88.01	87.33	87.37	—	24
**Transformer Network**	Deep Learning	35.25	26.87	35.25	20.05	—	24

## Data Availability

The raw data supporting the findings of this study are available from the corresponding author upon reasonable request.
